# Differences in the peripheral blood immune landscape between early-onset and late-onset colorectal cancer

**DOI:** 10.3389/fimmu.2025.1692382

**Published:** 2025-12-04

**Authors:** Clara Sánchez-Menéndez, Jaime Rodríguez-Pérez, Daniel Fuertes, Valentina Leguizamon, María González-Sanmartín, Elena Mateos, Miguel Cervero, Esther San José, Gonzalo Sanz, Edurne Álvaro, Araceli Ballestero-Pérez, Marc Martí-Gallostra, José Antonio Rueda, Elena Hurtado-Caballero, Carlos Pastor, Francesc Balaguer, Antonino Spinelli, Jorge Martínez-Laso, Montserrat Torres, José Perea, Mayte Coiras

**Affiliations:** 1 Immunopathology and Viral Reservoir Unit, National Center of Microbiology, Instituto de Salud Carlos III, Majadahonda, Madrid, Spain; 2 PhD Program in Biomedical Sciences and Public Health, Universidad Nacional de Educación a Distancia (UNED), Madrid, Spain; 3 Faculty of Biological Sciences, Universidad Complutense de Madrid, Madrid, Spain; 4 School of Telecommunications Engineering, Universidad Politécnica de Madrid, Madrid, Spain; 5 Department of Hematology and Hemotherapy, Instituto Ramón y Cajal de Investigación Sanitaria (IRYCIS), Hospital Universitario Ramón y Cajal, Madrid, Spain; 6 Faculty of Biological Sciences, Universidad de Alcalá, Madrid, Spain; 7 Biomedical Research Center Network in Infectious Diseases (CIBERINFEC), Instituto de Salud Carlos III, Majadahonda, Madrid, Spain; 8 School of Medicine, Universidad Alfonso X El Sabio, Madrid, Spain; 9 Department of Medicine, Faculty of Medicine, Health and Sports, Universidad Europea de Madrid, Madrid, Spain; 10 Surgery Department, San Carlos University Hospital, Madrid, Spain; 11 Surgery Department, Infanta Leonor University Hospital, Madrid, Spain; 12 Surgery Department, Ramon y Cajal University Hospital, Madrid, Spain; 13 Colorectal Unit, Vall d´Hebrón University Hospital, Universitat Autónoma de Barcelona (UAB), Barcelona, Spain; 14 Surgery Department, Alcorcon Foundation Hospital, Madrid, Spain; 15 Surgery Department, Gregorio Marañon University Hospital, Madrid, Spain; 16 Navarra University Clinic (Clínica Universidad de Navarra), Pamplona, Navarra, Spain; 17 Department of Gastroenterology, Hospital Clínic de Barcelona; Institut d’Investigacions Biomèdiques August Pi i Sunyer (IDIBAPS); Centro de Investigación Biomé dica en Red de Enfermedades Hepá ticas y Digestivas (CIBEREHD); University of Barcelona, Barcelona, Spain; 18 Department of Biomedical Sciences, Humanitas University, Pieve Emanuel, Milan, Italy. IRCCSHumanitas Research Hospital, Rozzano, Milan, Italy; 19 Immunogenetics Unit, National Center of Microbiology, Instituto de Salud Carlos III, Majadahonda, Madrid, Spain; 20 Biomedical Research Institute of Salamanca (IBSAL), Salamanca, Spain; 21 Coloproctology Unit, Hospital Universitario Vithas Madrid Arturo Soria, Madrid, Spain

**Keywords:** colorectal neoplasms, early diagnosis, immune response, T-cell subsets, cytokine profiling, immune biomarkers

## Abstract

**Introduction:**

Colorectal cancer (CRC) is a leading cause of cancer-related mortality. While screening has reduced incidence in older adults, cases of early-onset CRC (EOCRC), diagnosed before age 50, are rising, highlighting the need to understand its unique biology. Immune responses, particularly T-cell infiltration measured by the tumor-based Immunoscore, are known predictors of CRC prognosis, but less is known about systemic immune differences by age at diagnosis.

**Methods:**

Peripheral blood mononuclear cells (PBMCs) from EOCRC (n=19) and late-onset CRC (LOCRC; n=19) participants recruited in Madrid (Spain) were analyzed for immune cell phenotypes, exhaustion markers, soluble cytokines, and metabolic activity.

**Results:**

Our study revealed distinct peripheral blood immune profiles differentiating EOCRC from LOCRC. EOCRC patients exhibited a heightened proinflammatory environment, with increased functional capacity of CD4+ Th1, Th9, and Th17 subsets to produce IFNg, IL-9, and IL-17A, respectively, and increased plasma levels of IFNg and CXCL8/IL-8. This suggests an active but potentially ineffective immune response. Conversely, LOCRC patients showed hallmarks of immunosenescence and chronic inflammation, including impaired cytokine production, higher frequencies of CD8+ Tgd and Th22 cells, and increased plasma CCL13/MCP-4, consistent with tissue remodeling and immune suppression. Biomarkers distinguishing EOCRC included reduced Th22 and CD8+ Tgd cell frequencies and higher NKT-like cells with increased IL-13 production by Th22 cells.

**Conclusions:**

EOCRC and LOCRC involved different immune mechanisms, where EOCRC showed an altered proinflammatory environment with preserved regulatory pathways, while LOCRC reflected age-related immune decline and inflammaging. Peripheral blood immune profiling offers a minimally invasive liquid Immunoscore for early detection and enables personalized immunotherapies for age-related immune landscapes, particularly benefiting younger individuals at risk of EOCRC.

## 1 Introduction

Colorectal cancer (CRC) is the second most common cancer among women and the third most common among men in the Western world. In 2022, it accounted for 9.6% of all new cancer cases worldwide and was the second leading cause of cancer-related mortality, responsible for 9.3% of deaths (1). Widespread screening for precancerous lesions has led to a decline in the incidence of CRC in most Western countries since the 1990s, particularly among adults aged 65 and older (2, 3). However, this positive trend masks an alarming increase in early-onset CRC (EOCRC), defined as cases diagnosed before age 50.

CRC incidence in adults aged 40 to 49 years has increased by almost 15% since 2000 (4), and in 2017, 10.5% of new CRC cases were reported in people younger than 50 years (5). While CRC incidence has declined in people aged 65 and older since 2011, rates have remained steady in those aged 50–64 and have increased by about 2% per year in individuals under 50 and the 50–54 age group (3). The rising incidence of EOCRC is remarkably higher in some countries, such as the US or Canada, and in other regions, as in Europe, heterogenous patterns of EOCRC incidence have been reported (6, 7).

Multiple studies have demonstrated that EOCRC tumors are clinically and pathologically distinct from late-onset CRC (LOCRC). EOCRC patients present with higher rates of microsatellite instability (MSI), mainly due to mismatch repair deficiency (7–9), differences in CpG island methylation phenotype (7), unique DNA methylation patterns linked to aging (10), and altered chromosomal regions (11), with distinct chromosomal instability and mutation profiles (8, 12, 13). Moreover, EOCRC is unlikely related to inflammaging, the chronic low-grade inflammation associated with aging that characterizes LOCRC (11, 14). These biological and molecular differences may contribute to the distinct clinical behavior and diagnostic challenges observed in EOCRC. The positive association between common CRC symptoms and a later diagnosis is much stronger in individuals under 50 than in those over 50 (8, 9). However, the time between symptoms first appearing and diagnosis is still months long, due to low awareness of symptoms by primary care providers, which significantly reduces their possibilities of survival (10, 15). Therefore, the search for more accurate early diagnostic and prognostic biomarkers for EOCRC is currently a priority.

Present preventive and therapeutic approaches are increasingly focused on the tumor microenvironment and the development of an efficient anticancer immunity (16–18). Due to an overall stronger immune response correlates to better outcomes in cancer patients, high density of cytotoxic cells, memory cells, and tumor infiltrating T cells (TILs) are considered biomarkers of survival (16, 19). Therefore, a consensus Immunoscore has been developed as a standardized, reproducible assay that may accurately predict recurrence risk and overall survival in CRC by primarily quantifying mostly CD3+ and CD8+ T-cell infiltration in the tumor center and invasive margin (20). Patients with high Immunoscores showed lower risk of recurrence than those with low Immunoscores. Therefore, the marked differences between EOCRC and LOCRC have sparked interest in analyzing the role of the immune system in EOCRC development. However, because the Immunoscore relies on postoperative tumor specimens, its utility is restricted to prognosis after diagnosis rather than prevention or early detection, limitations particularly critical for EOCRC.

Despite the recognized importance of immune responses in CRC prognosis and the striking clinical differences between EOCRC and LOCRC, systemic immune profiles in peripheral blood have not been systematically compared between these age groups. Given that peripheral blood immune cells reflect systemic immune status and may provide accessible biomarkers, understanding how the circulating immune landscape differs between EOCRC and LOCRC could enable the development of minimally invasive diagnostic tools. A peripheral blood-based “liquid Immunoscore” could offer preventive and early-detection capabilities that the tissue-based Immunoscore cannot provide, potentially identifying high-risk individuals before tumors become clinically detectable.

This study aims to discover novel immune biomarkers specific to EOCRC that may permit the development of a non-invasive, peripheral blood-based “liquid Immunoscore” that may integrate specific quantitative measurements. By contrasting the immune landscapes of EOCRC and LOCRC based on key immune cell subsets, exhaustion markers, soluble cytokines, and metabolic capacity, we seek to improve early detection, enable more timely and personalized treatment, and ultimately identify individuals under 50 at high risk for EOCRC before tumors become clinically detectable.

## 2 Materials and methods

### 2.1 Participants

For this cohort study, participants diagnosed with EOCRC (n=19) were enrolled in Madrid (Spain) through the Spanish EOCRC Cohort (SECOC) (21). Patients with LOCRC (n=19) were also recruited for comparison. The inclusion criteria for EOCRC required participants to be over 18 years old but under 50 years old and have a confirmed diagnosis of CRC. Similarly, the inclusion criteria for LOCRC were to be over 50 years old. LOCRC cases were paired with EOCRC cases according to sex and tumor location. The exclusion criteria for both groups included having existing inflammatory bowel disease, histological diagnoses other than adenocarcinoma, and premalignant lesions/carcinomas *in situ*. All samples were collected before starting treatment, at the time of diagnosis. To rule out MSI status of the tumor, the presence of DNA mismatch repair (MMR) system deficiency was determined by immunohistochemistry, identifying the loss of the expression of any of the proteins MLH1, MSH2, MSH6, and PMS2 (22). To exclude hereditary forms, whole exome sequencing was performed, confirming that the microsatellite-stable (MSS) EOCRC cases analyzed were sporadic. Clinical and sociodemographic characteristics were collected for all participants.

### 2.2 Ethical statement

All individuals gave informed written consent to participate in the study before giving the blood sample. Protocol for this study (PIC 012-21-IIS-FJD) was prepared in accordance with the Helsinki Declaration and previously reviewed and approved by the Ethics Committee of Hospital Universitario Fundación Jiménez Díaz in Madrid (Spain). Current Spanish and European Data Protection Acts secured the confidentiality and anonymity of all participants.

### 2.3 Sample collection and processing

Blood samples were collected at the time of diagnosis before starting any treatment, either surgical or chemotherapy. The blood was immediately processed by centrifugation in a Ficoll-Hypaque density gradient (Corning, NY, USA) to isolate peripheral blood mononuclear cells (PBMCs) and plasma. After Ficoll separation, PBMCs were counted, assessed for viability, and cryopreserved at ≈10×10^6^ cells/mL in freezing medium (90% heat-inactivated FBS + 10% DMSO) using a controlled-rate cooling device (≈–1 °C/min ramp) before transfer to the vapor phase of liquid nitrogen for long-term storage. All assays were performed after a single freeze–thaw cycle. Thawing was carried out rapidly at 37 °C, followed by gradual dilution in pre-warmed culture medium and a short resting period before stimulation or phenotyping. To minimize batch effects, paired EOCRC–LOCRC samples were processed and analyzed in parallel whenever possible.

### 2.4 Cell lines

K562 cell line (ECACC 89121407) was generously provided by Dr Cristina Eguizabal (Basque Center of Transfusions and Human Tissues, Álava, Spain). Raji cell line (ECACC 85011429) was provided by the existing collection of the Instituto de Salud Carlos III (Madrid, Spain). Both cell lines were cultured in RPMI medium enriched with 10% fetal bovine serum (FBS), 100U/ml of penicillin/streptomycin, and 2mM of L-Glutamin (Lonza, Basel, Switzerland).

### 2.5 Immune phenotyping and quantification of cytokine production by CD4+ T helper subpopulations

To measure cytokines produced by CD4+ T helper (Th) cell subsets, PBMCs were stimulated with phorbol 12-myristate 13-acetate (PMA) (25ng/ml) and ionomycin (1.5µg/ml) for 4h at 37°C in the presence of brefeldin A (BD GolgiPlug, BD Biosciences) to block exocytotic transport through the Golgi complex, allowing the expressed cytokines to remain inside the cell (23). Cells were then stained with the following conjugated antibodies: CD3-PE, CD8-APCH7, CXCR3-BV421, CCR4-PECy7, CCR6-BV650, and CCR10-BUV395. CD4+ Th subsets (CD3+CD8-) were phenotyped according to the following patterns: Th1 (CXCR3+CCR6-), Th2 (CCR4+CCR6-), Th17 (CCR4+CCR6+), Th9 (CCR4-CCR6+), and Th22 (CCR4+CCR6+CCR10+). CD8+ T cells were also stained with the degranulation marker CD107a-PECy7 (BD Biosciences). After fixation and permeabilizing with IntraPrep Permeabilization Reagent (Immunostep, Salamanca, Spain), cells were intracellularly stained with the following antibodies to quantify the production of specific cytokines: IFNγ-FITC (Beckman Coulter, Brea, CA), IL4-APC, IL9-PercP, IL13-BV711, IL17a-BV510, and IL22-AF647 (BD Biosciences). Flow cytometry data were acquired using an LSRFortessa X-20 flow cytometer (BD Biosciences), and data analysis was performed using FlowJo v10.8 software (TreeStar Inc., Ashland, OR). The gating strategies used for the analysis of CD4+ Th subsets and their cytokine expression profiles are shown in **Supplementary Figures 1** and **2**, respectively.

### 2.6 Immune phenotyping of regulatory T cells

Regulatory T cells (Tregs) with the phenotype CD4+CD25+highCD127+low were characterized in PBMCs by flow cytometry using the following conjugated antibodies: CD4-PercP, CD25-PECy5, and CD127-FITC (BD Biosciences, San Jose, USA). Flow cytometry data acquisition and analysis were performed as described above.

### 2.7 Characterization of T-cell immune exhaustion and senescence

PBMCs were stained with Live/Dead Fixable Blue Dead Cell Stain Kit to discard dead cells and the following conjugated antibodies: CD3-NY660, CD4-BUV615, CD8a-NY730 (Thermo Fisher Scientific, Waltham, MA) to identify CD8+ and CD4+ T cells. Immune markers were stained as follows: senescence, CD57-PECyN7 and KLRG1-SB702; and exhaustion, PD1-SB780, LAG3-SB645, TIGIT-AF700, and TIM3-APC (Thermo Fisher Scientific). Flow cytometry data were acquired using a Cytek Aurora flow cytometer and SpectroFlo software (Cytek Biosciences, Fremont, CA), and data analysis was performed using FlowJo v10.8 software (TreeStar Inc.). The gating strategy used for the analysis of CD4+ and CD8+ T cell immune senescence and exhaustion is shown in **Supplementary Figure 3**.

### 2.8 Immune phenotyping and quantification of cytokine production by NK and NKT-like cells

NK and NKT-like cells, with the phenotype CD3-CD56+ and CD3+CD56+, respectively, were phenotyped based on the expression of activation and inhibition markers on their cell surface, using the following antibodies (BD Biosciences): inhibitory receptors, NKG2A-PE and CD158f-BV421; activating receptors, NKG2C-AF700, NKG2D-PECy7, NKp44-BUV395, and NKp46-BV650.

To measure cytokines produced by NK and NKT-like cells, PBMCs were stimulated with 1µg/ml of Hsp70 peptide (Abcam, Cambridge, UK) at 37°C in the presence of Brefeldin A (BD Biosciences) for 4 hours. Cells were then stained with the following conjugated antibodies: CD3-APC, CD56-BV605, CD16-BV421, CD107a-PECy7, and TCRγδ-BUV395. After fixation and permeabilization with IntraPrep Permeabilization Reagent (Immunostep), cells were intracellularly stained with IFNγ-PE (Beckman Coulter, Brea, CA), TNFα-PE, and Granzyme B (GZB)-FITC (BD Biosciences). Flow cytometry data acquisition and analysis were performed as described above. The gating strategy used for the phenotyping of NK and NKT cells and their cytokine expression profiles is shown in **Supplementary Figure 4**. The gating strategy to analyze CD8+ T cells and Tgd cells is shown in **Supplementary Figure 5**.

### 2.9 Measurement of capacity for direct cellular cytotoxicity of NK cells

Direct cellular cytotoxicity (DCC) of NK cells was measured as previously reported (24) using K562 cell line as missing-self target cells (25). K562 cells were stained with PKH26 Red Fluorescence Cell Linker kit (Sigma Aldrich-Merck) and then co-cultured for 1 hour at 37°C with PBMCs (1:1). Cells were then collected and Annexin V conjugated with FITC (Thermo Fisher) was used to measure early apoptosis by flow cytometry in stained K562 cells using an LSRFortessa X-20 flow cytometer (BD Biosciences). Data analysis was performed using FlowJo V10.8 software (TreeStar Inc.).

### 2.10 Measurement of glucose uptake in immune cell populations

To measure the capacity of PBMCs to uptake glucose, cells were incubated in glucose-deficient RPMI medium for 2h at 37°C. The fluorescent derivative of D-glucose monomer 2-[N-(7-nitrobenz-2-oxa-1,3-diazol-4-yl)amino]-2-deoxy-d-glucose (2-NBDG) (26), was added to the culture medium and incubated for 10 min at 37°C. Antibody against GLUT-1 conjugated with AF647 (BD Biosciences) was used to evaluate the expression of this marker. Flow cytometry data were acquired using an LSRFortessa X-20 flow cytometer (BD Biosciences), and data analysis was performed using FlowJo v10.8 software (TreeStar Inc.). The gating strategy used for the analysis of 2-NBDG probe uptake and Glut-1 expression is shown in **Supplementary Figure 6**.

### 2.11 Cytokine and chemokine levels in plasma

A custom 21-plex Human Magnetic Luminex Assay kit (R&D Systems, Minnesota, USA) was used to quantify the following cytokines in plasma samples: pro-inflammatory, IL-1α, IL-1β, IL-6, IL12p70, IL-17, IFNβ, IFNγ, and TNFα; anti-inflammatory, IL-1RA, IL-4, and IL-10; chemokines, CCL2/MCP-1, CCL3/MIP-1α, CCL4/MIP-1β, CCL13/MCP-4, CCL20/MIP-3α, CCL23/MPIF, CXCL8/IL-8, and CXCL9/MIG; regulatory or homeostatic, IL-2, IL-7, and IL-15. A Luminex 200 system was used to acquire and analyze results using xPONENT software (Thermo Fisher Scientific).

### 2.12 Principal component analysis

Linear dimensionality reduction was performed using principal component analysis (PCA) (27) to capture the global structure of the data by finding the directions that maximize variance in the data. Python 3.11 was used with Pandas (28) for data import, NumPy (29) for numerical operations, Scikit-learn (30) for data normalization (in range [0, 1]) of continuous variables, and one-hot encoding for categorical features. Only parameters showing statistical significance (p<0.05) in univariate testing were analyzed. A biplot illustrating group clusters was generated with Matplotlib (31).

### 2.13 Random forest

A Random Forest algorithm (32) was applied to predict the categorization of participants with EOCRC or LOCRC and evaluate the resulting accuracy. The selection of these parameters was performed according to the existence of significant statistical differences between groups (p<0.05). To avoid bias in the selection of training, testing and validation sets, we performed a combined feature selection and classification procedure using a Random Forest classifier with a nested 5-fold cross-validation procedure for each competing algorithm, as previously described (33, 34). The relative importance for each feature in the categorization of participants with EOCRC or LOCRC was calculated by the Gini VIM method (35).

### 2.14 Statistical analysis

Statistical analysis was performed with GraphPad Prism v10.2.1 (GraphPad Software Inc.) and STATA 14.2 software (StataCorp LLC, College Station, TX). Samples’ normal distribution was tested using the Shapiro-Wilk test. Quantitative variables were described as the median and interquartile range (IQR) and qualitative variables as absolute or relative frequencies. Qualitative data were compared by Fisher´s exact test, and quantitative data using the student’s t-test or Mann-Whitney U test, as appropriate. Associations between qualitative clinical data and age of CRC development were assessed using binary logistic regression analysis (odds ratio, OR) and 95% confidence interval (CI). Associations between quantitative parameters and age of CRC development were determined using simple and logistic regressions to estimate the OR and CI, comparing LOCRC data as reference versus EOCRC data. To analyze data correlation and compute the Spearman coefficient r between all Th subsets per CRC cohort and within each cohort, we applied a combination of Python libraries such as Scikit-Learn (36) and Pandas (28, 37). For the generation of regression plots, the Seaborn library was used (38). Values of p<0.05 were considered statistically significant in all comparisons.

## 3 Results

### 3.1 Study population

This observational study recruited two cohorts of participants with CRC stratified by age (n=38). Sociodemographic and clinical descriptions of the participants are summarized in **Table 1** and described in more detail in **Supplementary Table 1**. The EOCRC cohort had a median age of 44 years (IQR 41-48), while the LOCRC cohort had a median age of 76 years (IQR 55-84). Over half of the participants in both cohorts were men (63.2% in EOCRC and 52.6% in LOCRC). In all analyses, samples from men and women were identified separately to determine whether sex-related differences may exist in any of the assessed parameters with potential influence on the development of CRC.

**Table 1:** Sociodemographic and clinical data of all participants in the study.

	EOCRC (n=19)	LOCRC (n=19)	P value
Age at cancer diagnosis, years; median (IQR)	44 (41–48)	76 (55–84)	**<0.0001**
Sex; n. male (%)	12 (63.2)	10 (52.6)	0.7431
Cancer location			
Right colon, n. (%)	5 (26.4)	6 (31.6)	1
Left colon, n. (%)	7 (36.8)	8 (42.1)	1
Rectum, n. (%)	7 (36.8)	5 (26.3)	0.7281
Stage			
I, n. (%)	3 (15.8)	5 (26.3)	0.6928
II, n. (%)	5 (26.3)	7 (36.8)	0.7281
III, n. (%)	6 (31.6)	7 (36.8)	1
IV, n. (%)	5 (26.3)	0 (0)	**0.0463**
Microsatellite instability, n. (%)	3 (15.8)	0 (0)	0.2297
CRC family history			
FDR, n. (%)	7 (36.8)	Unk.	–
SDR, n. (%)	2 (10.5)	Unk.	–
Sporadic, n. (%)	10 (52.6)	Unk.	–
Main comorbidities			
DM, n. (%)	2 (10.5)	4 (21.1)	0.6599
DL, n. (%)	2 (10.5)	4 (21.1)	0.6599
HTN, n. (%)	0 (0)	10 (52.6)	**0.0004**
Heart conditions, n. (%)	0 (0)	5 (26.3)	**0.0463**
Respiratory conditions, n. (%)	2 (10.5)	4 (21.1)	0.6599
Main treatment at sample collection			
Lipid lowering medication, n. (%)	2 (10.5)	7 (36.8)	0.1245
Anticoagulants, n. (%)	1 (5.3)	7 (36.8)	**0.0422**
Antihypertensive medication, n. (%)	0 (0)	8 (42.1)	**0.0031**
Diabetes medication, n. (%)	2 (10.5)	3 (15.8)	1

CRC, Colorectal cancer; DM, Diabetes mellitus; DL, Dyslipidemia; FDR, First degree relative; HTN, Hypertension; IQR, Interquartile Range; SDR, Second degree relative; Unk., Unknown. Fisher´s exact test was used to calculate statistical differences between cohorts. Significant p-values are highlighted in bold.

Most participants presented with tumors in the left colon (36.8% in EOCRC and 42.1% in LOCRC), predominantly at stage III (31.6% in EOCRC and 36.8% in LOCRC), with no statistically significant differences between cohorts. Five participants in EOCRC cohort (26.3%) presented stage IV versus none in LOCRC cohort. MSI analysis revealed loss of protein expression in 3 participants (15.8%) in the EOCRC cohort, while none was observed in the LOCRC cohort. Family history of CRC was assessed only in EOCRC cases and showed that 52.6% were classified as sporadic. Whole exome sequencing confirmed that the MSS EOCRC cases included in our analyses were not associated with known hereditary CRC syndromes, ensuring that the comparisons reflect sporadic EOCRC versus LOCRC.

Comorbidities at the time of cancer diagnosis were more frequent in the LOCRC cohort (89.5% vs. 42.1% in EOCRC), with hypertension and heart conditions being the most prevalent (52.6% and 26.3%, respectively). Similarly, 84.2% of participants in the LOCRC cohort were undergoing medical treatment at the time of sampling, compared to 36.8% in the EOCRC cohort, with antihypertensive medication being the most common treatment in LOCRC (42.1%).

A binary logistic regression analysis was performed to evaluate whether demographic, clinical, and treatment variables were associated with the occurrence of EOCRC. None of these variables reached statistical significance, except for treatment with anticoagulants, which was associated with a higher odds ratio (OR = 10.50; 95% CI = 1.14–96.58; p=0.038) (**Supplementary Table 2**).

### 3.2 Levels of CD4+ T cells and expression of dysfunction markers were similar between cohorts

**Figure 1:**
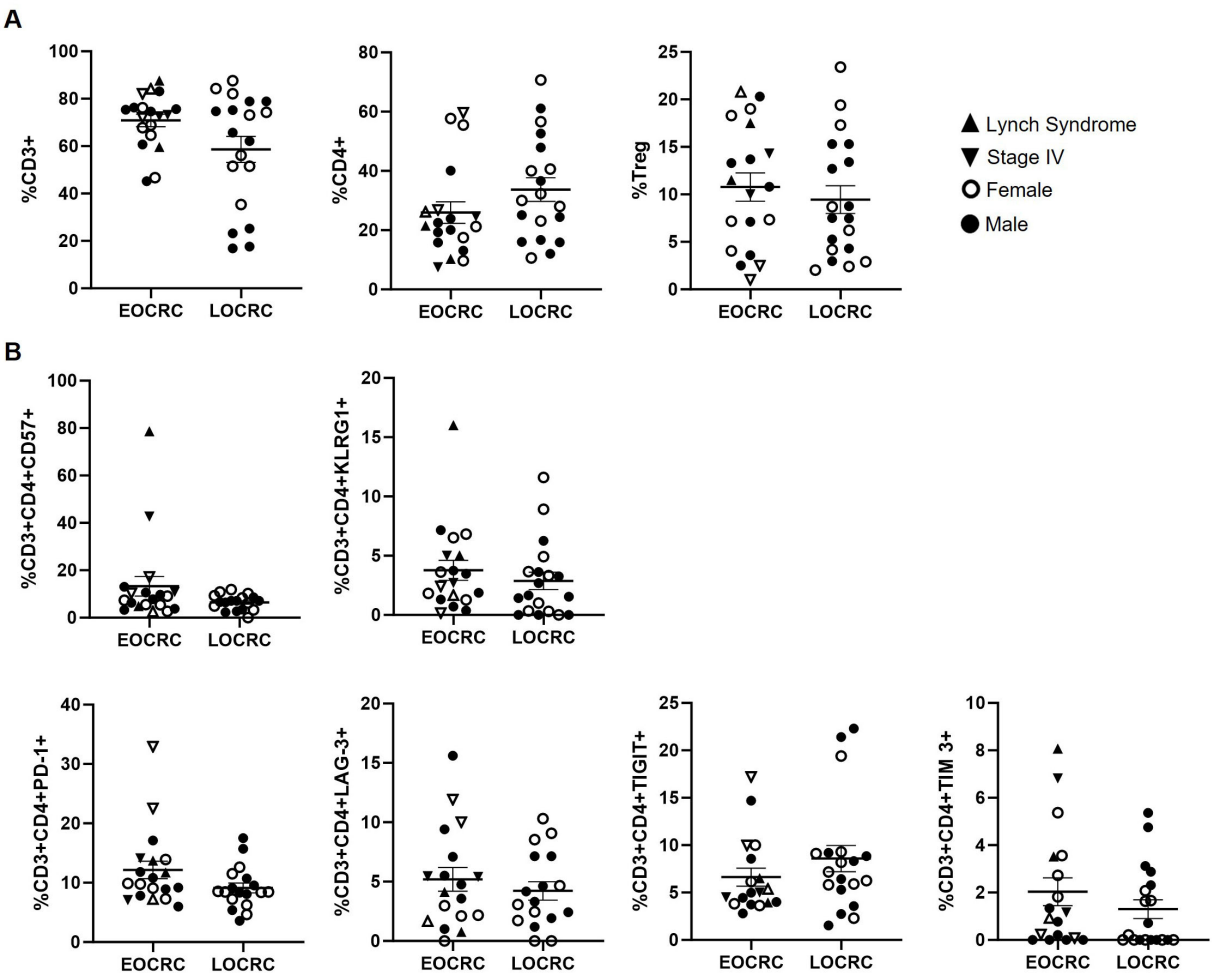
Peripheral blood CD4+ T cell composition and immune aging markers in EOCRC and LOCRC participants. **(A)** Levels of total T cells (CD3+), CD4+ T cells, and Tregs in peripheral blood of EOCRC and LOCRC participants. **(B)** Expression of immune senescence markers and exhaustion markers in CD4+ T cells from the participants. Each symbol corresponds to one sample and vertical lines represent the standard error of the mean (SEM). Open symbols correspond to EOCRC and closed symbols correspond to LOCRC. Upright closed triangles indicate participants with Lynch syndrome and inverted closed triangle indicate participants with stage IV. Statistical analysis was performed with Mann-Whitney test or Unpaired t test as appropriate.

We found no significant differences in the levels of total lymphocytes (CD3+), CD4+ T cells, or Tregs between the EOCRC and LOCRC cohorts (**Figure 1A**). Similarly, the expression of immune senescence markers (CD57 and KLRG1) and exhaustion markers (PD-1, LAG-3, TIGIT, and TIM-3) in CD4+ T cells did not differ significantly between cohorts (**Figure 1B**).

### 3.3 Enhanced functionality of CD4+ Th1 and Th2 cells in EOCRC participants

**Figure 2:**
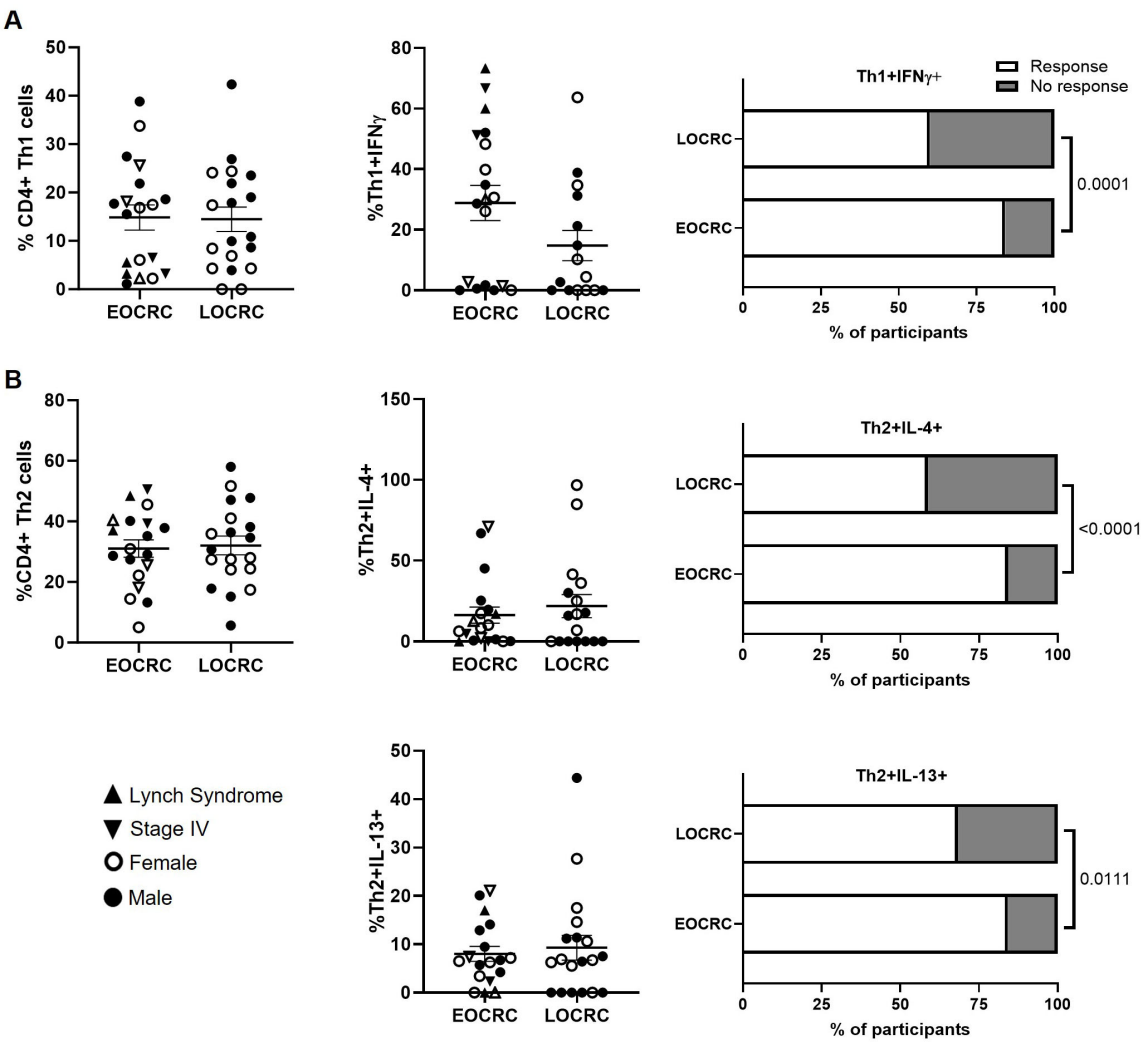
Functional profiling of peripheral CD4+ Th1 and Th2 cells in EOCRC and LOCRC participants. **(A)** Frequency of CD4+ Th1 cells in peripheral blood of EOCRC and LOCRC and levels of IFNγ produced by these cells. **(B)** Frequency of CD4+ Th2 cells and levels of IL-4 and IL-13 produced by these cells. Bar graphs on the right represent the percentage of cells able to produce each cytokine. Open symbols correspond to EOCRC and closed symbols correspond to LOCRC. Upright closed triangles indicate participants with Lynch syndrome and inverted closed triangle indicate participants with stage IV. Statistical analysis was performed with Mann-Whitney test or Unpaired t test as appropriate. Fisher´s exact test was used to calculate significance between cohorts in horizontal bar graphs.

The levels of CD4+ Th1 cells were comparable between the EOCRC and LOCRC cohorts (**Figure 2A**, left graph). No significant differences were observed in the levels of IFNγ produced by these cells (**Figure 2A**, center graph); however, 40% of participants in the LOCRC cohort had CD4+ Th1 cells that were unable to produce IFNγ, compared to 15.8% in the EOCRC cohort (p=0.0001) (**Figure 2A**, right graph).

Similarly, CD4+ Th2 cell levels were comparable between the cohorts (**Figure 2B**, left graph), as were the levels of IL-4 and IL-13 produced by these cells (**Figure 2B**, center graphs). Nevertheless, 41.2% of LOCRC participants had Th2 cells with a reduced capacity to produce IL-4, compared to 15.8% in EOCRC (p<0.0001). Likewise, 31.6% of LOCRC participants and 15.8% of EOCRC participants showed a reduced capacity to produce IL-13 (p=0.0111) (**Figures 2B**, right graphs).

The calculation of Spearman’s correlation between both cohorts in the levels of CD4+ Th1 and Th2 and representative cytokines produced by these cells showed no significant correlation between groups (**Supplementary Figure 7A**). However, within the EOCRC cohort, there was a moderate negative Th1/Th2 correlation (r=-0.45; p=0.05) (**Supplementary Figure 7B**), while this expected inverse association was absent in LOCRC (**Supplementary Figure 7C**).

### 3.4 Enhanced Th9/Th17 cytokine production but reduced Th22 cell levels in EOCRC

**Figure 3:**
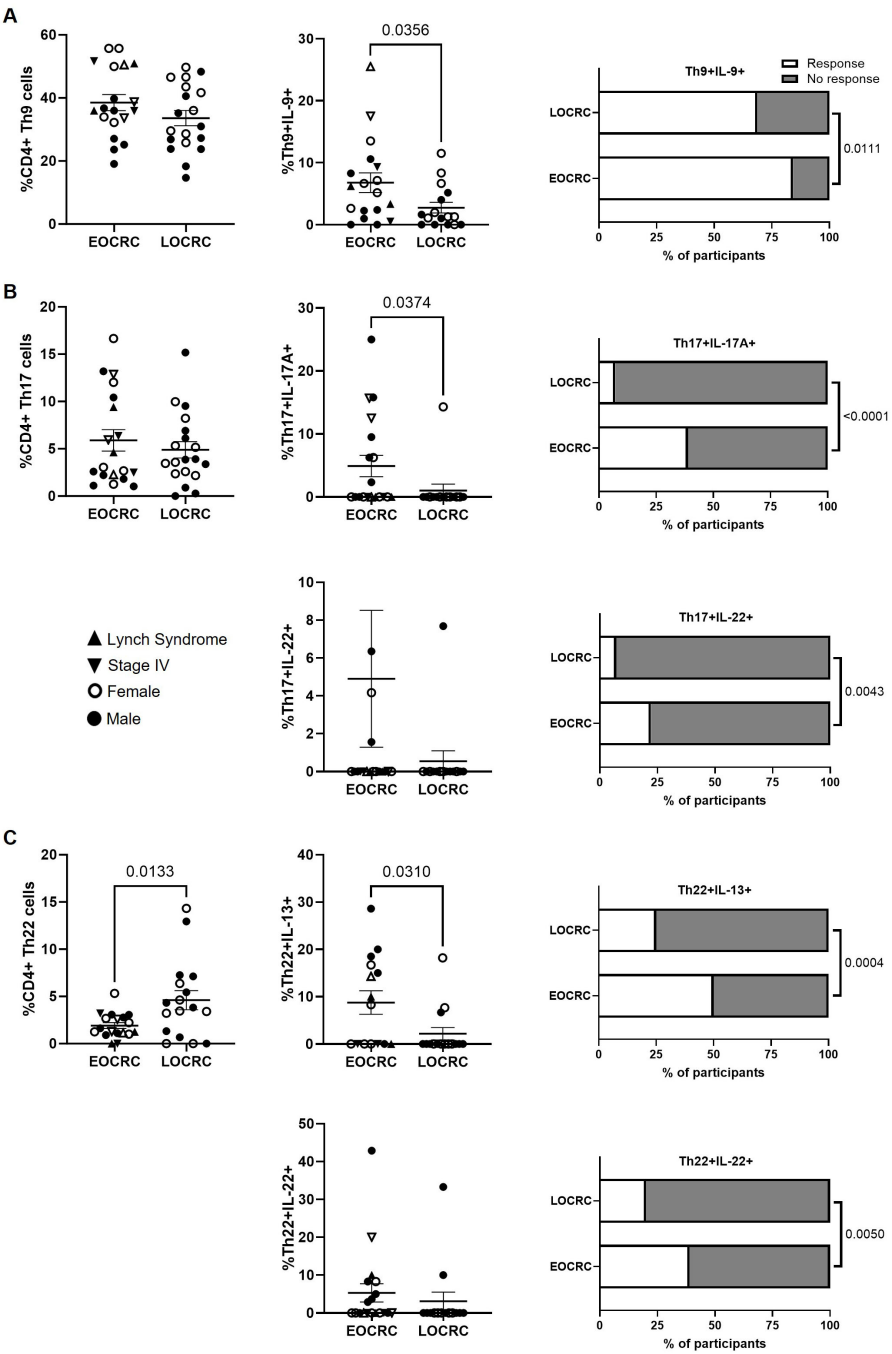
Functional profiling of peripheral CD4+ Th9, Th17, and Th22 cells in EOCRC and LOCRC participants. **(A)** Frequency of CD4+ Th9 cells in peripheral blood of EOCRC and LOCRC and levels of IL-9 produced by these cells. **(B)** Frequency of CD4+ Th17 cells and levels of IL-17A and IL-22 produced by these cells. **(C)** Frequency of CD4+ Th22 cells and levels of IL-13 and IL-22 produced by these cells. Bar graphs on the right represent the percentage of cells able to produce each cytokine. Open symbols correspond to EOCRC and closed symbols correspond to LOCRC. Upright closed triangles indicate participants with Lynch syndrome and inverted closed triangle indicate participants with stage IV. Statistical analysis was performed with Mann-Whitney test or Unpaired t test as appropriate. Fisher´s exact test was used to calculate significance between cohorts in horizontal bar graphs.

The levels of CD4+ Th9 cells were comparable between the EOCRC and LOCRC cohorts (**Figure 3A**, left graph), but cells from EOCRC participants produced significantly higher levels of IL-9 than those of the LOCRC cohort (2.5-fold; p=0.0356) (**Figure 3A**, center graph). Notably, 31.3% of LOCRC participants had Th9 cells that were unable to produce IL-9, compared to 15.8% in the EOCRC cohort (**Figure 3A**, right graph).

Similarly, CD4+ Th17 cell levels did not differ between the cohorts (**Figure 3B**, left graph), nor did the levels of IL-22 produced by these cells; however, Th17 cells from participants of EOCRC produced higher levels of IL-17A than LOCRC (4.8-fold; p=0.0374) (**Figure 3B**, center graphs). Moreover, a reduced capacity to produce IL-17A was observed in Th17 cells from 92.2% of LOCRC participants, compared to 61.1% in EOCRC (p<0.0001). Likewise, 92.2% of LOCRC participants and 77.8% of EOCRC participants showed impaired IL-22 production (p=0.0043) (**Figures 3B**, right graphs).

CD4+ Th22 cell levels were 2.4-fold lower in EOCRC participants compared to those in LOCRC (p=0.0133) (**Figure 3C**, left graph). In contrast, IL-13 levels produced by these cells were 4-fold higher compared to LOCRC (p=0.0310), while IL-22 production did not differ significantly between cohorts (**Figure 3C**, center graphs). However, impaired IL-13 production was observed in Th22 cells from 75% of LOCRC participants, compared to 50% in EOCRC (p = 0.0004). Similarly, 80% of LOCRC participants and 61.1% of EOCRC participants showed reduced IL-22 production capacity (p=0.0050) (**Figures 3C**, right graphs).

The calculation of Spearman’s correlation between both cohorts in the levels of CD4+ Th9, Th17, and Th22 and representative cytokines produced by these cells showed no significant correlation between groups (**Supplementary Figure 7A**). There was a significant negative association between Th9 and Th22 in the EOCRC cohort (r = -0.53, p = 0.02) (**Supplementary Figure 7B**), contrasted by distinct patterns in LOCRC: a significant negative correlation between Th1 and Th22 (r = -0.55, p = 0.02), a borderline association between Th2 and Th9 (r = -0.44, p = 0.06), and a moderate positive correlation between Th17 and Th22 (r = 0.49, p = 0.05) (**Supplementary Figure 7C**).

### 3.5 CD8+ T cells from EOCRC showed similar levels to LOCRC but higher LAG-3 expression

**Figure 4:**
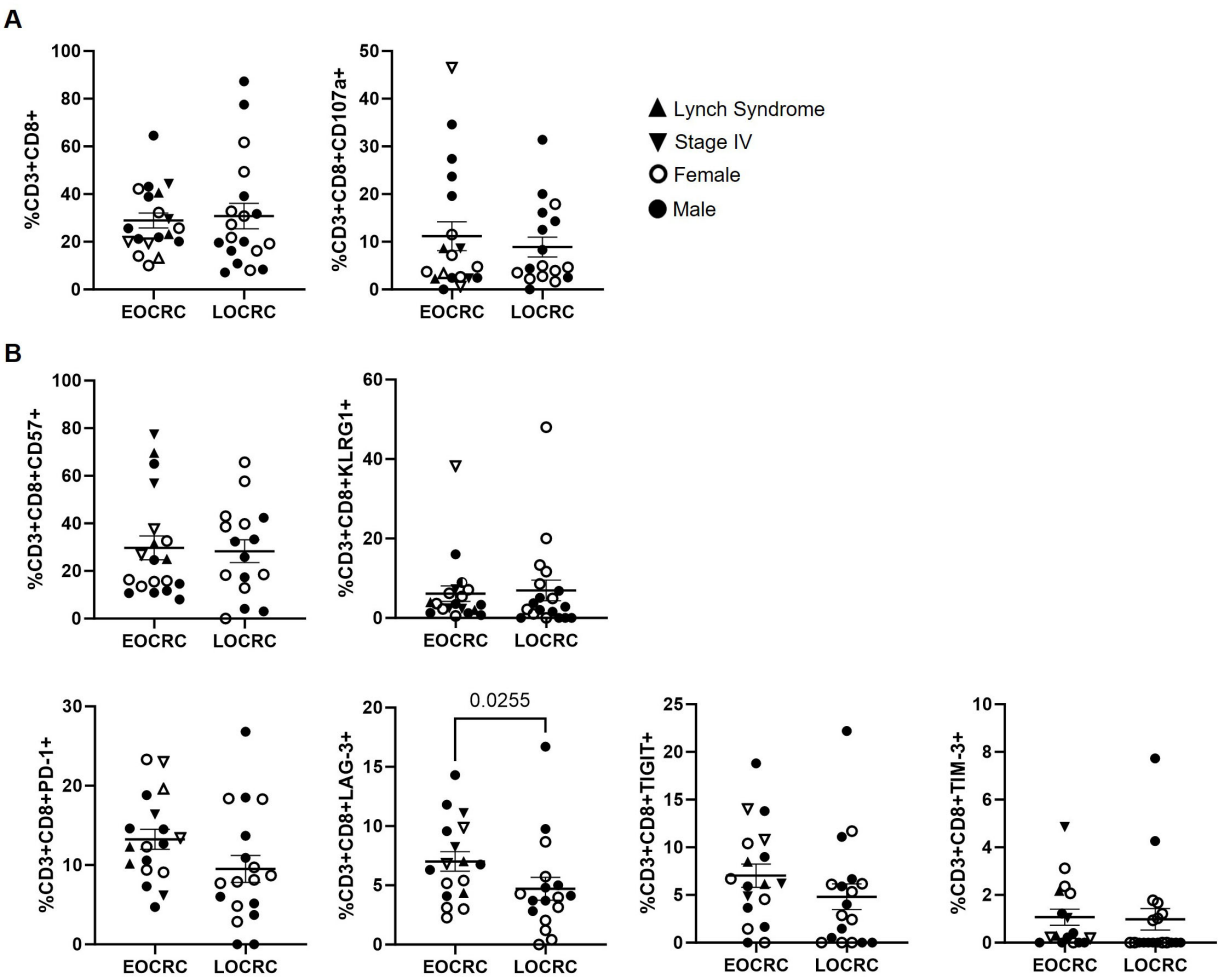
Peripheral blood CD8+ T cell composition and immune aging markers in EOCRC and LOCRC participants. **(A)** Levels of total CD8+ T cells (left graph) and expression of the degranulation marker CD107a (right graph) in peripheral blood of EOCRC and LOCRC participants. **(B)** Expression of immune senescence markers and exhaustion markers in CD8+ T cells from the participants. Each symbol corresponds to one sample and vertical lines represent the standard error of the mean (SEM). Open symbols correspond to EOCRC and closed symbols correspond to LOCRC. Upright closed triangles indicate participants with Lynch syndrome and inverted closed triangle indicate participants with stage IV. Statistical analysis was performed with Mann-Whitney test or Unpaired t test as appropriate.

No differences were observed between cohorts in CD8+ T cell levels or in their degranulation capacity, assessed by CD107a expression (**Figure 4A**). The analysis of immune senescence and exhaustion markers in these cells revealed a significantly higher expression of the exhaustion marker LAG-3 in EOCRC participants, with a 1.5-fold increase compared to LOCRC (p=0.0255) (**Figure 4B**).

### 3.6 Reduction of CD8+ Tγδ cells in EOCRC with preserved degranulation capacity

**Figure 5:**
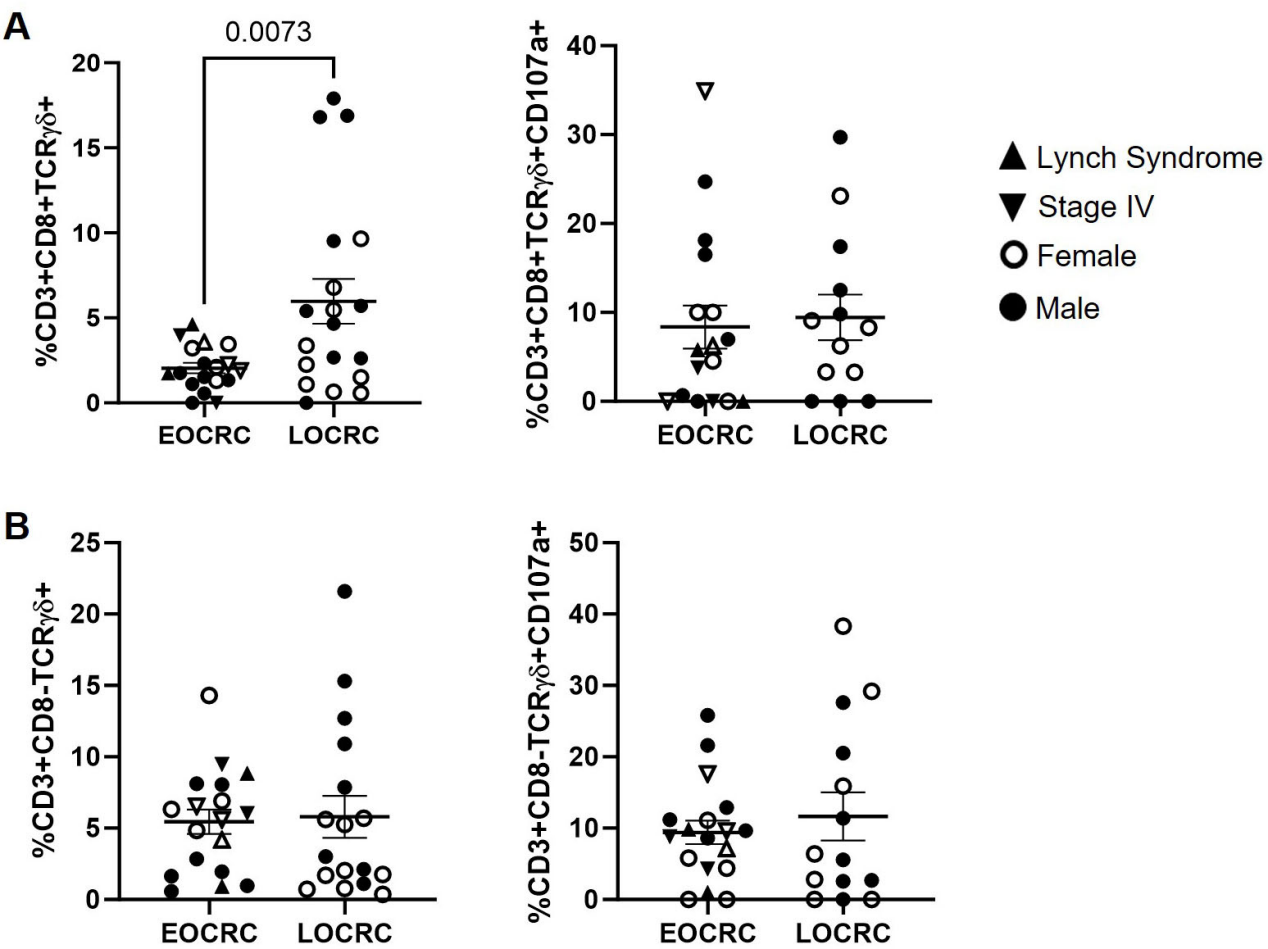
Peripheral blood Tγδ cell levels and expression of CD107a in EOCRC and LOCRC participants. Levels of CD8+ **(A)** and CD8- **(B)** Tγδ cells (left graphs) and expression of the degranulation marker CD107a in these cells (right graphs) in peripheral blood of EOCRC and LOCRC participants. Each symbol corresponds to one sample and vertical lines represent the standard error of the mean (SEM). Open symbols correspond to EOCRC and closed symbols correspond to LOCRC. Upright closed triangles indicate participants with Lynch syndrome and inverted closed triangle indicate participants with stage IV. Statistical analysis was performed with Mann-Whitney test or Unpaired t test as appropriate.

CD8+ Tγδ cell levels were 2.9-fold lower in EOCRC compared to LOCRC (p=0.0073), while CD107a expression in these cells was similar between cohorts (**Figure 5A**). No differences were observed between cohorts in the levels of CD8- Tγδ cells or in their CD107a expression (**Figure 5B**).

### 3.7 Higher NKT-like cell levels in EOCRC with comparable functionality

**Figure 6:**
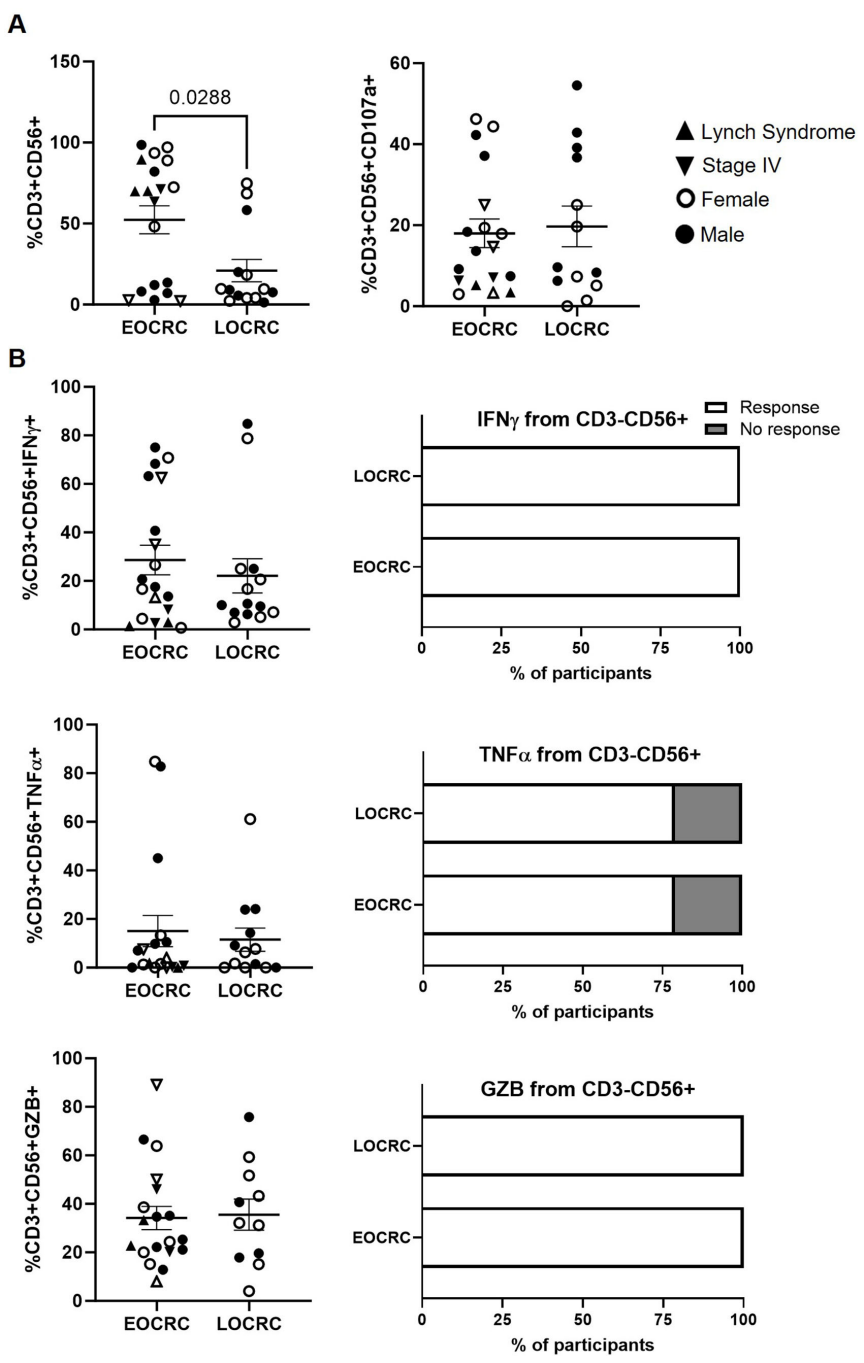
Functional profiling of peripheral NKT-like cells in EOCRC and LOCRC participants. **(A)** Levels of NKT-like cells in peripheral blood of EOCRC and LOCRC and expression of CD107a in these cells. **(B)** Levels of IFNγ, TNFα, and GZB produced by NKT-like cells. Bar graphs on the right represent the percentage of cells able to produce each cytokine. Open symbols correspond to EOCRC and closed symbols correspond to LOCRC. Upright closed triangles indicate participants with Lynch syndrome and inverted closed triangle indicate participants with stage IV. Statistical analysis was performed with Mann-Whitney test or Unpaired t test as appropriate. Fisher´s exact test was used to calculate significance between cohorts in horizontal bar graphs.

NKT-like cell levels were 2.5-fold higher in EOCRC compared to LOCRC (p=0.0288) (**Figure 6A**, left graph). However, no differences were observed in their degranulation capacity (**Figure 6A**, right graph) or in cytokine production in response to Hsp70 peptides (**Figure 6B**).

### 3.8 NK cells in EOCRC showed similar phenotypic profiles but reduced cytotoxic capacity

**Figure 7:**
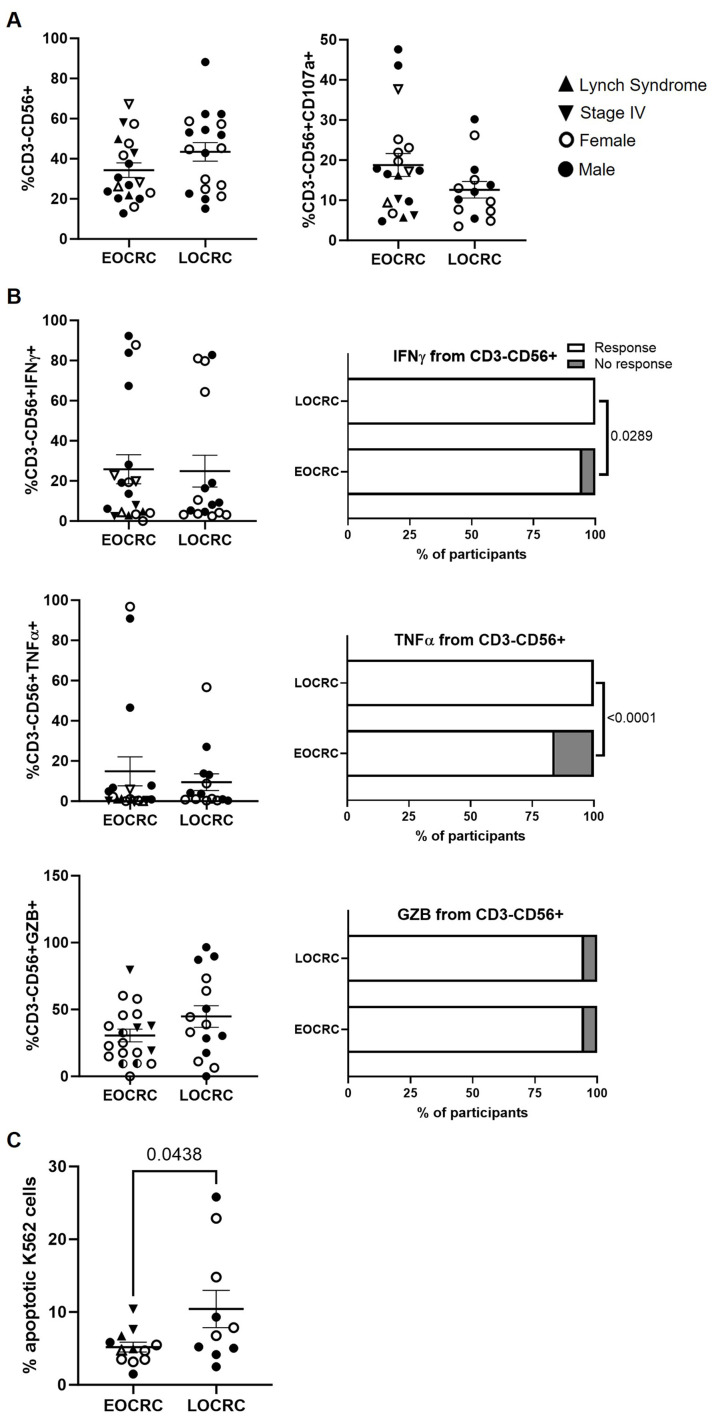
Functional profiling of peripheral NK cells in EOCRC and LOCRC participants. **(A)** Levels of NK cells in peripheral blood of EOCRC and LOCRC and expression of CD107a in these cells. **(B)** Levels of IFNγ, TNFα, and GZB produced by NK cells. Bar graphs on the right represent the percentage of cells able to produce each cytokine. **(C)** Percentage of K562 cells in apoptosis after co-culture with PBMCs from the participants. Open symbols correspond to EOCRC and closed symbols correspond to LOCRC. Upright closed triangles indicate participants with Lynch syndrome and inverted closed triangle indicate participants with stage IV. Statistical analysis was performed with Mann-Whitney test or Unpaired t test as appropriate. Fisher´s exact test was used to calculate significance between cohorts in horizontal bar graphs.

NK cell levels and their degranulation capacity were similar between cohorts (**Figure 7A**). Although there were no significant differences in the levels of cytokines produced by these cells in response to Hsp70 peptides (**Figure 7B**, left graphs), a proportion of EOCRC participants presented NK cells lacking the capacity to produce IFNγ (5.3%, p=0.0289) and TNFα (15.8%; p<0.0001) (**Figure 7B**, right graphs). The capacity of these cells to exert direct cellular cytotoxicity (DCC) against K562 target cells was 2.0-fold reduced in EOCRC compared to LOCRC (p=0.0438) (**Figure 7C**).

We found no differences between groups in the expression of inhibitory or activating markers on the NK cell surface (**Supplementary Figure 8**).

### 3.9 Higher glucose uptake in EOCRC PBMCs with unchanged GLUT-1 expression

**Figure 8:**
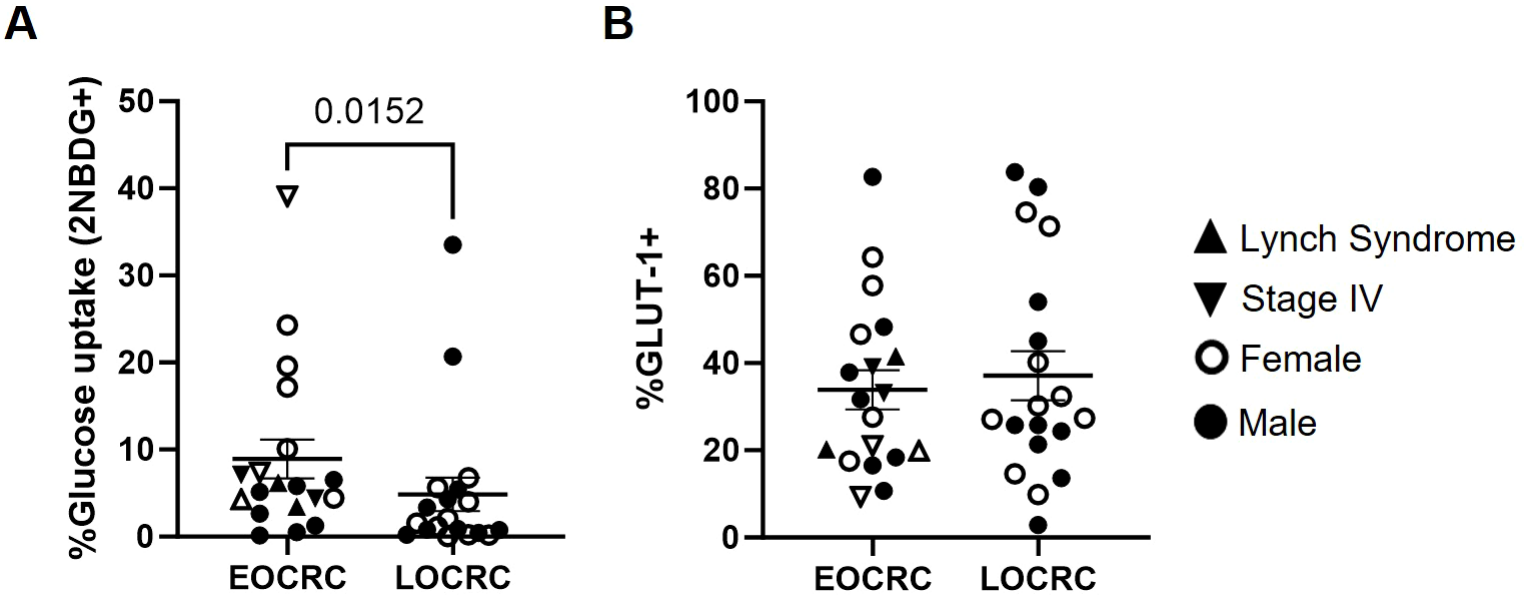
Glucose uptake and GLUT-1 expression in PBMCs from EOCRC and LOCRC participants. Percentage of uptake of the glucose analog 2-NBDG by PBMCs from the participants **(A)** and expression of GLUT-1 receptor on the surface of these cells **(B)**. Open symbols correspond to EOCRC and closed symbols correspond to LOCRC. Upright closed triangles indicate participants with Lynch syndrome and inverted closed triangle indicate participants with stage IV. Statistical analysis was performed with Mann-Whitney test or Unpaired t test as appropriate.

PBMCs from EOCRC participants showed 1.8-fold higher glucose uptake than LOCRC (p=0.0152), as measured using the glucose analog 2-NBDG (**Figure 8A**). No differences between cohorts were observed in the expression levels of GLUT-1 transporter (**Figure 8B**).

### 3.10 Increased systemic inflammatory markers IFNγ and IL-8/CXCR8 in EOCRC

**Figure 9:**
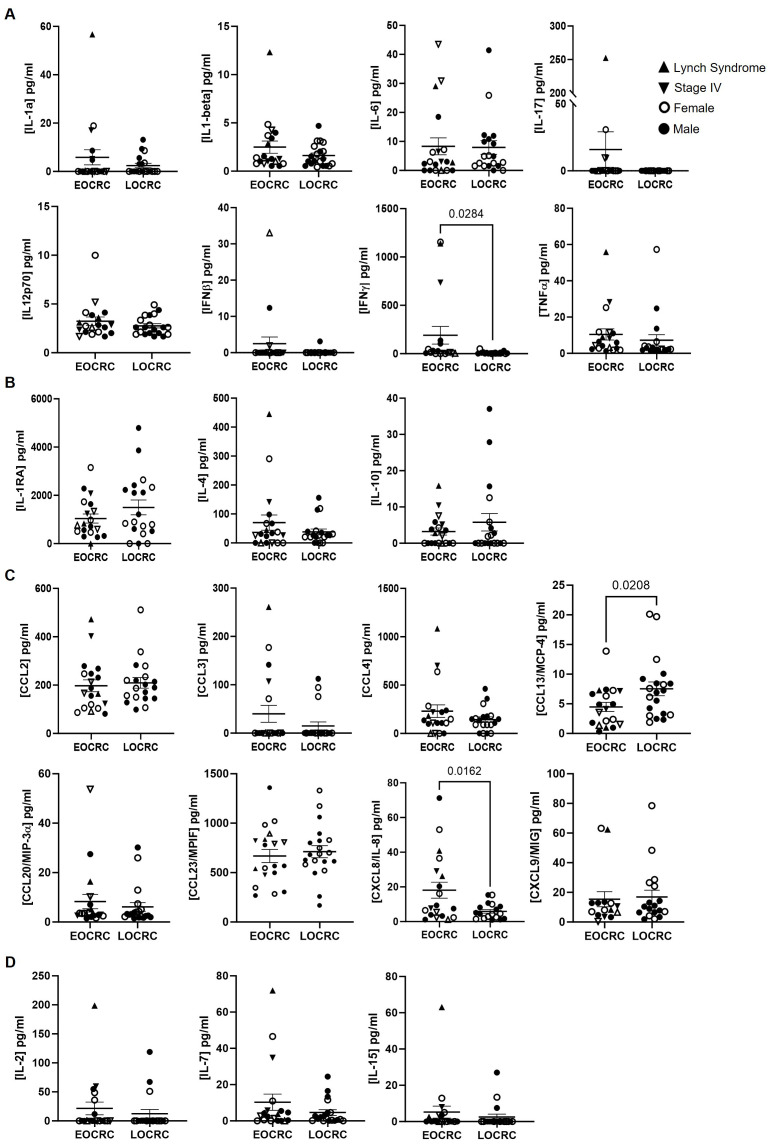
Plasma cytokine levels in EOCRC and LOCRC participants. Levels of pro-inflammatory **(A)**, anti-inflammatory **(B)**, chemotactic **(C)**, and homeostatic **(D)** cytokines in plasma of the participants. Open symbols correspond to EOCRC and closed symbols correspond to LOCRC. Upright closed triangles indicate participants with Lynch syndrome and inverted closed triangle indicate participants with stage IV. Statistical analysis was performed with Mann-Whitney test or Unpaired t test as appropriate.

Plasma cytokine analysis revealed significantly higher levels of IFNγ (23-fold; p=0.0284) and CXCR8/IL-8 (3.2-fold; p= 0.0162) in EOCRC participants compared to LOCRC, along with lower levels of CCL13/MCP-4 (-1.7-fold; p=0.0208) (**Figure 9**).

### 3.11 Distinct immunometabolic profiling signatures differentiated EOCRC from LOCRC

**Figure 10:**
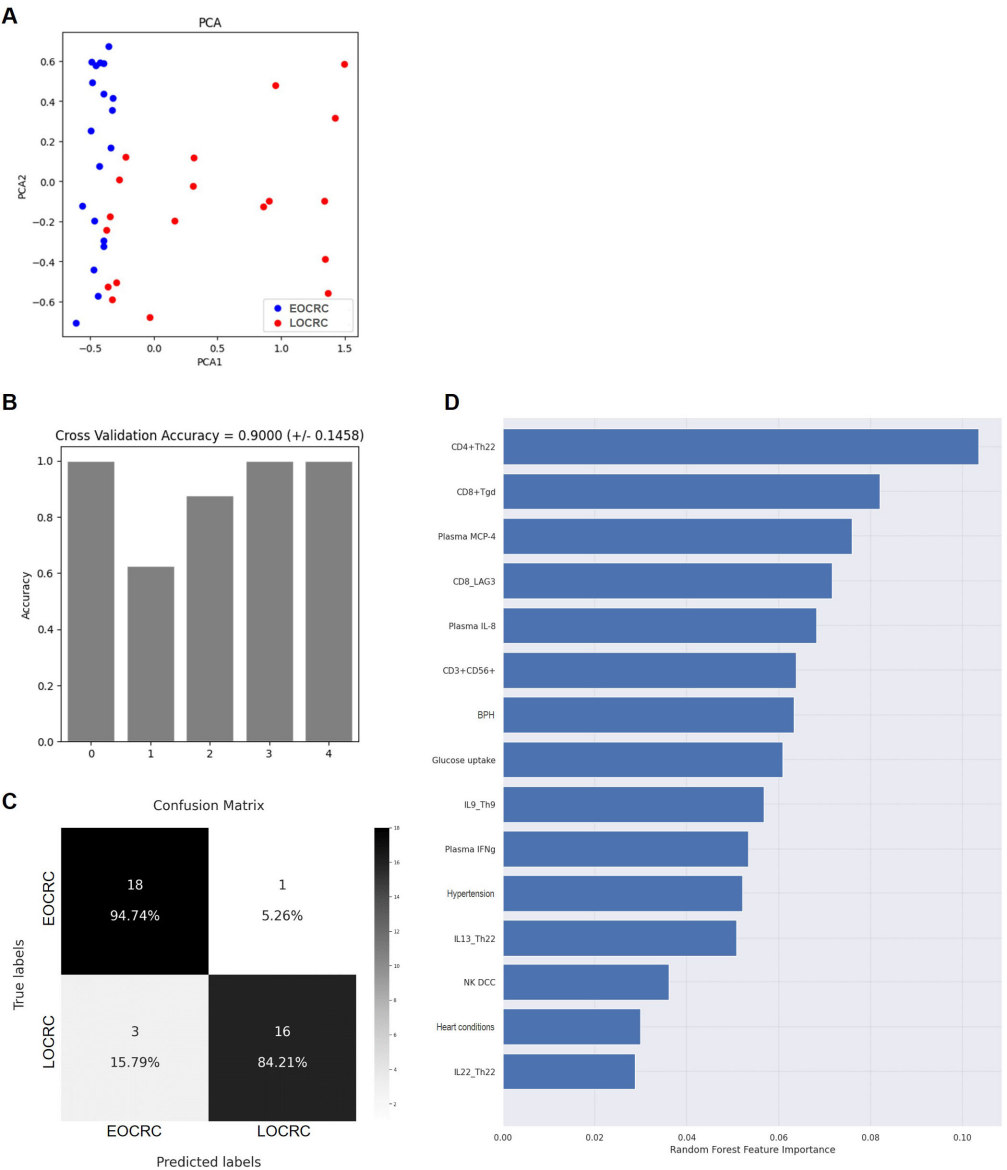
PCA visualization and random forest-based classification performance and feature importance of selected immune biomarkers. **(A)** PCA-based visualization showing separation of EOCRC and LOCRC participants. **(B)** Classification accuracy across 5 outer iterations of nested K-fold cross-validation using a random forest model. **(C)** Confusion matrix comparing predicted and actual diagnostic categories (EOCRC vs. LOCRC). **(D)** Relative importance of selected biomarkers for classification based on the Gini variable importance measure. BPH, Benign prostatic hyperplasia; DCC, Direct cellular cytotoxicity; IFNγ, Interferon gamma; LAG-3, Lymphocyte-activation gene 3; MCP-4, Monocyte chemoattractant protein-4.

PCA revealed a partial separation between EOCRC and LOCRC participants along the first principal component (PCA1) (**Figure 10A**). Although there was some overlap, most EOCRC samples clustered on the left side of the PCA1 axis, while LOCRC samples displayed a broader distribution across both PCA1 and PCA2 axes. These results suggest that the multivariate immune parameters contributing to PCA1, potentially related to immune, metabolic, or molecular parameters, differed between both cohorts, with EOCRC individuals exhibiting a more homogeneous profile.

To assess whether immunological and metabolic parameters could discriminate between EOCRC and LOCRC, we trained a Random Forest classifier using selected variables. The model achieved a mean cross-validation accuracy of 90.0% (± 14.6%) across five folds (**Figure 10B**), with a confusion matrix showing high classification performance: 94.7% accuracy for EOCRC and 84.2% for LOCRC (**Figure 10C**). The most informative features contributing to classification were counts of CD4+ Th22 cells, CD8+ Tγδ cells, and CD8+ T cells expressing the exhaustion marker LAG-3, as well as plasma levels of CCL13/MCP-4 (**Figure 10D**). These results suggested that a distinct immunometabolic signature can effectively distinguish between EOCRC and LOCRC.

### 3.12 Immunological predictors of risk for EOCRC

Most of the discriminative features identified by the random forest model were corroborated by binary logistic regression, which identified four parameters with significant predictive value (**Supplementary Table 3**): CD4+ Th22 cells (β=0.0638, OR = 1.5012, p=0.034), CD8+ TCRγδ+ cells (β=0.0479, OR = 1.4861, p=0.035), IL-13 produced by Th22 cells (β=-0.0250, OR = 0.8835, p=0.044), and NKT-like cells (β=-0.0060, OR = 0.9722, p=0.019).

## 4 Discussion

CRC remains a leading cause of cancer-related death worldwide. While screening programs have successfully reduced incidence in older adults, the occurrence of EOCRC has risen sharply over the past decades (12, 39), challenging paradigms and fundamental biological differences between age-stratified diseases. The tissue-based Immunoscore has proven a superior predictor of disease-free and overall survival compared to the conventional TNM (Tumor Nodes Metastasis) staging system (40). However, because it relies on postoperative tumor specimens, its utility is limited to prognosis after diagnosis rather than prevention or early detection. Whether the immune determinants of prognosis identified in tumor tissue are reflected in peripheral blood, and whether they differ fundamentally between EOCRC and LOCRC, remains unexplored. This study addresses these questions by comprehensively profiling circulating immune landscapes in age-stratified CRC cohorts to identify biomarkers suitable for a minimally invasive liquid Immunoscore.

In our study, CRC participants were stratified by age into EOCRC and LOCRC, without statistically significant differences between cohorts in cancer stages I-III, location, or MSI. Notably, five EOCRC individuals had stage IV disease versus none in the LOCRC cohort. Although this imbalance could confound immune-profile comparisons, these five stage-IV cases did not cluster separately in any of our analyses, indicating that the immune signatures we identified are driven by age-related factors rather than metastatic burden. Whole exome sequencing confirmed that the MSS EOCRC cases included in our analyses were not associated with known hereditary CRC syndromes, ensuring that the comparisons presented here reflect sporadic EOCRC versus LOCRC. Additionally, even though 89.5% of LOCRC participants presented comorbidities versus 42.1% of EOCRC participants, and most LOCRC participants were taking medication to treat these comorbidities, these percentages were similar to those observed in the general population for this age group (41, 42). Furthermore, although we considered sex in the analysis of each parameter, no clear pattern emerged to suggest that sex plays a major role in the development of EOCRC. This was further supported by the odds ratio analysis, which indicated that sex was not associated with an increased risk. These findings validate that the distinct immune landscapes we report are genuine age-associated phenomena rather than artefacts of clinical confounders.

Total peripheral blood immune cell counts such as CD3+, CD4+, and regulatory T cells, as well as markers of T-cell senescence/exhaustion in total CD4+ populations, did not differ between EOCRC and LOCRC cohorts. However, functional assays revealed an increased capacity to produce cytokines from the different CD4+ Th subsets in participants with EOCRC, who also presented a distinctly more proinflammatory Th1/Th9/Th17 profile. This functional activation pattern, rather than reflecting immune decline, is consistent with the immune profile typically observed in younger individuals, characterized by stronger Th polarization and preserved naïve/memory balance (43). Such features may indicate that EOCRC arises in a proinflammatory systemic milieu, whereas LOCRC reflects features of immunosenescence and “inflammaging,” with reduced adaptive flexibility and impaired effector function (44).

An adequate CD4+ Th cell polarization is essential for the appropriate coordination of cellular and humoral responses against pathogens or cancer cells (45, 46), and each subset contributes differently to tumor progression. In CRC, a preferential polarization to Th1 intracellular response is generally assumed to promote prolonged disease-free survival (47–49), mostly due to their capacity to produce IFNγ that may activate an efficient response against intracellular challenges (50). CD4+ Th1 cells from EOCRC participants showed higher capacity to produce IFNγ, whose plasma levels were significantly increased compared to the LOCRC cohort. This finding contrasts with previous reports (49) and studies in advanced CRC showing impaired IFNγ responses associated with immune exhaustion and tumor progression. Importantly, our EOCRC cohort, despite including five stage IV cases, maintained robust IFNγ production, suggesting that age-related immune competence may override stage-dependent exhaustion mechanisms in younger patients. This is further supported by recent single-cell analyses, which show that younger patients retain higher proportions of functional effector T cells, even in advanced disease stages (51). These results suggested that the immune response of younger participants could be better at controlling tumor progression. In fact, the classical, mutually inhibitory Th1/Th2 axis was only preserved in EOCRC participants, thereby reflecting intact IFNγ-mediated suppression of Th2 differentiation (52). However, the absence of significantly negative correlation in LOCRC indicates that aging may disrupt the normal balance between opposing Th cell subpopulations.

CD4+ Th17 subset has been associated with tumor progression by inducing IL-17-mediated angiogenesis, suppressing CD8+ TILs, and thereby promoting a worse prognosis (49, 53–55). However, Th17 cells may also recruit neutrophils and CD8+ T cells directly via chemokines like CXCL8/IL-8, which has been linked to better CRC outcomes (56). Therefore, Th17 cells may present a dual role in CRC (56). Our EOCRC participants showed higher capacity to produce IL-17A from Th17 cells than LOCRC, which aligns with studies describing inflammatory mucosal signatures and Th17-enriched microenvironments in early-onset tumors (57), along with higher plasma levels of CXCL8/IL-8. This profile might reflect an inflammatory but ineffective immune response, insufficient for full elimination of tumor cells yet capable of promoting angiogenesis and early tumor growth. Conversely, LOCRC patients exhibited elevated plasma levels of CCL13/MCP-4, a chemokine involved in the recruitment of monocytes, eosinophils, and Th2-skewed lymphocytes that mediate tissue remodeling and chronic inflammation (58), likely reflecting a pro-remodeling, Th2-biased immune environment consistent with immunosenescence and inflammaging. The concurrent decrease in IFNγ and IL-8 further supports a defective Th1-mediated effector function and impaired inflammatory signaling, indicating a shift from protective anti-tumor immunity toward chronic, dysregulated inflammation. This cytokine signature is in line with inflammaging processes that favor tissue repair and remodeling over immune clearance, thereby potentially facilitating tumor persistence in LOCRC.

Similarly, CD4+ Th9 cells have also shown a dual effect on tumors (59), presenting anti-tumorigenic effects in most cancers (60, 61), but able to promote CRC in colitis-associated neoplasm models (62). The frequencies of Th9 cells in the CRC tumor positively correlate with the rates of CD8+ TILs (63). Although we did not observe significant differences between cohorts in the Th9 cell count, these cells expressed higher levels of IL-9 in EOCRC participants than LOCRC. Due to IL-9 may amplify IL-17-driven inflammation and angiogenesis (64) and stimulate both Th17 activity and CXCL8/IL-8 production (65), this cytokine strongly contributes to the general pro-inflammatory environment observed in EOCRC participants. This coordinated upregulation of IL-9 and IL-17A may indicate a proangiogenic, early inflammatory state favoring tumor initiation, as suggested in models of colitis-associated CRC (66). Conversely, LOCRC patients, with higher IL-13-producing Th22 cells, exhibited a reparative, senescent-like immune signature potentially associated with chronic inflammation and tissue remodeling rather than acute immune activation (67).

Finally, the levels of CD4+ Th22 cells were significantly reduced in our EOCRC cohort. These cells are characterized by the production of IL-22 and also exhibit a dual role in CRC, acting in tissue repair in both early-stage disease and tumor progression as the inflammation becomes chronic (68). IL-22 has been involved in mucosal defense, tissue repair, and wound healing (69), but also with CRC tumor progression through the activation of the STAT3 pathway, promoting cancer cell self-renewal and tumorigenesis (70). Intriguingly, while Th22 frequencies were reduced in EOCRC, these cells showed enhanced per-cell capacity to produce both IL-22 and IL-13 compared to LOCRC. This functional enhancement despite numerical reduction suggests a compensatory activation phenotype, where fewer cells attempt to maintain tissue homeostasis under inflammatory stress. The elevated IL-13 production by Th22 cells in EOCRC is particularly notable, as IL-13 is typically produced by CD4+ Th2 cells, involved in promoting tissue remodeling and fibrosis (71). The elevated production of IL-13 by Th22 cells in EOCRC patients suggests a potential shift in their functional profile, which together with their reduced levels, may promote a proinflammatory environment with an impaired capacity for tissue repair and immune regulation in EOCRC, potentially leading to unchecked tumor progression. However, Th22 cells also showed enhanced capacity to produce IL-22 and IL-13 in EOCRC, which could promote aggressive tumor behavior and shape an immunosuppressive environment favorable to tumor survival and growth (72). In contrast, LOCRC patients exhibited an accumulation of Th22 cells with impaired capacity to produce IL-22, which supported a dysregulated immune response that may contribute to chronic inflammation and tumor development. This pattern aligns with findings showing that aged immune systems accumulate phenotypically defined but functionally exhausted cell subsets (73), contributing to chronic inflammation without effective tissue repair, a state conducive to tumor development and progression.

Moreover, Th9/Th22 correlation was negative in EOCRC cohort, suggesting that IL-9-driven inflammatory signals may help restrain IL-22-mediated STAT3 activation and tumor-promoting self-renewal pathways (62, 74). By contrast, LOCRC participants exhibited a broad reduction in cytokine production across multiple Th subsets, highlighted by negative Th1/Th22 and Th2/Th9 correlations, as well as a positive Th17/Th22 correlation, thereby pointing at a functional decline in adaptive immunity. This altered polarization may reflect immunosenescent and inflammaging phenotypes characterized by skewing toward IL-22/IL-17-driven tissue repair and chronic inflammation (68, 72, 75). Consistent with our observation of a higher proportion of non-IFNγ-producing Th1 and reduced cytokine production in Th2 cells in LOCRC, age-associated declines in type-1 T cell function and reduced IFNγ production have been repeatedly documented in older individuals (76, 77). These changes reflect hallmarks of immunosenescence in LOCRC and are associated with reduced adaptive immunity and chronic, low-grade inflammation.

Additionally, LOCRC participants showed higher frequency of CD8+ Tγδ cells that under chronic inflammatory or senescent conditions may adopt regulatory or tissue-repair functions instead of exerting direct cytotoxic activity against tumor cells (78). This suggests that despite the preserved numbers, the cytotoxic potential of these cells is functionally compromised in LOCRC, contributing to impaired tumor immunosurveillance and a shift toward a pro-remodeling, chronic inflammatory microenvironment. Collectively, cytotoxic cell phenotype and CD4+ Th subset interactions may imply that EOCRC pathogenesis involves a failure of immune elimination despite preserved regulatory pathways and increased levels of NKT-like cells in peripheral blood, which was supported by the reduced cytotoxic capacity of NK cells and likely NKT cells, and by increased levels of exhausted LAG3+ CD8+ T cells. However, LOCRC seems to arise from progressive immune decline induced by inflammaging and loss of tumor immunosurveillance, illustrated by an overall consistent impaired functional and metabolic capacity in the immune cells. Specifically, the combination of higher regulatory-like CD8+ Tγδ cells and reduced NK/NKT cytotoxicity reflects a functional decline in the effector arm of immunity, pointing to a mechanistic link between immunosenescence and reduced anti-tumor activity in older patients.

In our study, PBMCs from EOCRC patients displayed higher glucose uptake than those from LOCRC, despite no differences in GLUT-1 expression. This observation may indicate a global enhancement of metabolic activity among circulating mononuclear cells. Previous studies have shown that immune activation and systemic inflammation are accompanied by metabolic remodeling of PBMCs, including increased glycolytic flux and altered mitochondrial function in diverse settings (79, 80), and systemic inflammatory responses have been documented in CRC (81). Moreover, studies measuring circulating immune bioenergetics in cancer patients indicate that PBMC metabolic phenotypes can reflect disease-associated immune states (82). We speculate that the increased metabolic activity of immune cells in EOCRC may favor the emergence of highly glycolytic tumor clones, potentially contributing to their more aggressive clinical behavior. In contrast, LOCRC PBMCs showed reduced metabolic activity, which may limit cytokine production and cytotoxic responses, contributing to chronic inflammation and tissue-remodeling milieu characteristic of immunosenescence.

Feature importance analysis ranked Th22 cell frequency, CD8+ Tγδ cells, plasma CCL13/MCP-4, and LAG3+ CD8+ T cells as the top contributors to age-based CRC stratification. These findings were corroborated by binary logistic regression, which indicated that decreased frequencies of Th22 cells and CD8+ Tγδ cells were risk factors for EOCRC, suggesting an altered proinflammatory environment with functionally compromised immune responses that fail to contain early tumor progression. Conversely, higher IL-13 production by Th22 cells and increased circulating NKT-like cells were protective factors for EOCRC, indicating compensatory immunoregulatory and innate-like antitumor activity more prominent in early-onset disease. In contrast, LOCRC participants exhibited broader variation across these immune-metabolic parameters, consistent with immunosenescence, reduced cytotoxic capacity, and impaired effector function.

The main limitation of this study was that although peripheral blood is an accessible source for immune monitoring, circulating immune phenotypes may not fully represent the tumor microenvironment, where local immune landscape and cell-cell interactions may modulate effector function. Therefore, further integrating tumor microenvironment analyses would be necessary to deepen understanding of these systemic immune alterations.

In conclusion, our findings revealed distinct immunological profiles characterizing EOCRC versus LOCRC. EOCRC presented a heightened proinflammatory environment with altered functionality but retaining compensatory regulatory pathways. In contrast, LOCRC exhibited features consistent with immunosenescence and inflammaging, with impaired cytokine production and a shift toward tissue-remodeling and chronic inflammation pathways. These findings highlight the potential to incorporate differential biomarkers such as frequencies and functional states of Th22 cells, CD8+ Tγδ cells, IL-13 production, and NKT-like cells into a liquid Immunoscore based on peripheral blood analysis. Unlike classical Immunoscore, which relies on postoperative tumor tissue and serves primarily a prognostic role, a liquid Immunoscore would offer a minimally invasive, accessible tool with preventive capabilities. This could enable early immune-based risk stratification and detection of CRC, particularly in younger populations at risk for EOCRC, facilitating timely and personalized immunotherapeutic interventions to improve clinical outcomes. Further studies with larger cohorts would be needed to ensure the validity of the approach.

## Data Availability

The original contributions presented in the study are included in the article/**Supplementary Material**. Further inquiries can be directed to the corresponding authors.

## Group members of the Spanish EOCRC consortium

José A. Alcazar¹, Oscar Alonso², Carlos Álvarez Laso^3^, Edgardo Celi Altamarina^4^, Alicia Alvarellos^5^, Edurne Álvaro^6^, Jorge Arredondo^5^, María Arriba Domenech^7^, Francesc Balaguer^8,9,10,11^, Araceli Ballestero^14^, Javier Barambio^6^, Francisco Blanco Antona¹, Lorena Brandáriz^6^, Ana Burdaspal^6^, Sabela Carballal^8,9,10,11^, Joaquín Castillo^8,9,10,11^, Adriana Cavero^12^, Cristian Cisterne^4^, Gonzalo Colmenarejo^13^, Miriam Cuatrecasas^8,9,10,11^, María Daca-Alvarez^8,9,10,11^, Javier Die Trill^14^, Jana Dziakova^2^, Isabel Espinosa-Salinas^13^, Lidia Estudillo^15^, Lara P. Fernández^13^, Alba Fernández Candela^16^, José M. Fernández Cebrián^14^, Daniel Fernández Martínez^3^, María Luisa de Fuenmayor^6^, Leire García Alonso^3^, Luis García Florez^3^, Elena García García^4^, Juan L. García^17^, Damián García-Olmo^18^, Ariadna García-Rodríguez^19^, Marta Gómez de Cedrón^13^, Rogelio González-Sarmiento^17^, Pablo Granero Castro^3^, Pol Guarner Piquet^20^, Sergio Hernández-Villafranca^18^, Elena Hurtado^7^, Fernando Jiménez Escovar^12^, Luis M. Jiménez Gómez^7^, Marta Jiménez Toscano^21^, Miquel Kraft^19^, Hardeep Kumari^8,9,10,11^, Ignacio Hevia Lorenzo^3^, Ana B. Herrero^17^, Mar Iglesias Coma^21^, Irene López Rojo^2^, Carmen Martínez Sánchez^20^, Núria Malats^15^, Franco Marinello^19^, Marc Martí^19^, Ignacio Matos^5^, Leticia Moreira^8,9,10,11^, Lorena Moreno^8,9,10,11^, Paula Munoz Munoz^16^, Juan Ocana Jiménez^14^, Teresa Ocana^8,9,10,11^, Ma del Mar Pardo^4^, Carlos Pastor^5^, Isabel Peligros Gómez^6^, María Pellisé^8,9,10,11^, José Perea^17,23^, Sara Picazo Marín^22^, Vicente Portugal^12^, Jésica Pérez^17^, Ana Ramírez de Molina^13^, Javier Rodríguez^5^, José I. Rodríguez García^3^, José A. Rueda Orgaz^4^, Gonzalo Sanz^22^, Rodrigo Sanz López^22^, Ariadna Sánchez^8,9,10,11^, Aida Suárez Álvarez^3^, María Suárez Solís^22^, Ana Teijo^2^, Nuria Truan Alonso^3^, Laura Vega López^4^, Rosario Vidal Tocino¹, Ignacio Valverde^24^, Cristina Viyuela^24^, Jaime Zorrilla Ortúzar^7^.

^1^Salamanca University Hospital, Salamanca, Spain

^2^MD Anderson Cancer Center Spain, Madrid, Spain

^3^Central de Asturias University Hospital, Oviedo, Spain

^4^Fundación Hospital Alcorcón, Alcorcón, Madrid, Spain

^5^Navarra University Clinic (Clínica Universidad de Navarra), Pamplona, Navarra, Spain

^6^Infanta Leonor University Hospital, Madrid, Spain

^7^Gregorio Maranón University Hospital, Madrid, Spain

^8^Department of Gastroenterology, Hospital Clınic de Barcelona, Barcelona, Spain

^9^Institut d'Investigacions Biomediques August Pi i Sunyer (IDIBAPS), Barcelona, Spain

^10^Centro de Investigación Biomédica en Red de Enfermedades Hepáticas y Digestivas (CIBEREHD), Barcelona, Spain

^11^University of Barcelona, Barcelona, Spain

^12^Galdakao-Usansolo Hospital, Galdakao, Vizcaya, Spain

^13^Molecular Oncology IMDEA Food Institute, Madrid, Spain

^14^Ramón y Cajal University Hospital, Madrid, Spain

^15^Spanish National Cancer Research Center (CNIO), Genetic & Molecular Epidemiology Group, Madrid, Spain

^16^Quirónsalud Torrevieja Hospital, Torrevieja, Alicante, Spain

^17^Biomedical Research Institute of Salamanca (IBSAL), Salamanca, Spain

^18^Fundación Jiménez Díaz University Hospital, Madrid, Spain

^19^Vall d'Hebron University Hospital, Barcelona, Spain

^20^Hospital de la Santa Creu i Sant Pau. Barcelona, Spain

^21^Hospital del Mar, Barcelona, Spain

^22^Hospital Universitario Clínico San Carlos, Madrid, Spain

^23^Vithas Arturo Soria University Hospital, Madrid, Spain

^24^Villalba University Hospital, Madrid, Spain
