# Correction: Differences in the peripheral blood immune landscape between early-onset and late-onset colorectal cancer

**DOI:** 10.3389/fimmu.2026.1833566

**Published:** 2026-04-06

**Authors:** Clara Sánchez-Menéndez, Jaime Rodríguez-Pérez, Daniel Fuertes, Valentina Leguizamon, María González-Sanmartin, Elena Mateos, Miguel Cervero, Esther San José, Gonzalo Sanz, Edurne Álvaro, Araceli Ballestero-Pérez, Marc Martí-Gallostra, José Antonio Rueda, Elena Hurtado-Caballero, Carlos Pastor, Francesc Balaguer, Antonino Spinelli, Jorge Martínez-Laso, Montserrat Torres, José Perea, Mayte Coiras

**Affiliations:** 1 Immunopathology and Viral Reservoir Unit, National Center of Microbiology, Instituto de Salud Carlos III, Majadahonda, Madrid, Spain; 2 PhD Program in Biomedical Sciences and Public Health, Universidad Nacional de Educación a Distancia (UNED), Madrid, Spain; 3 Faculty of Biological Sciences, Universidad Complutense de Madrid, Madrid, Spain; 4 School of Telecommunications Engineering, Universidad Politécnica de Madrid, Madrid, Spain; 5 Department of Hematology and Hemotherapy, Instituto Ramón y Cajal de Investigación Sanitaria (IRYCIS), Hospital Universitario Ramón y Cajal, Madrid, Spain; 6 Faculty of Biological Sciences, Universidad de Alcalá, Madrid, Spain; 7 Biomedical Research Center Network in Infectious Diseases (CIBERINFEC), Instituto de Salud Carlos III, Majadahonda, Madrid, Spain; 8 School of Medicine, Universidad Alfonso X El Sabio, Madrid, Spain; 9 Department of Medicine, Faculty of Medicine, Health and Sports, Universidad Europea de Madrid, Madrid, Spain; 10 Surgery Department, San Carlos University Hospital, Madrid, Spain; 11 Surgery Department, Infanta Leonor University Hospital, Madrid, Spain; 12 Surgery Department, Ramon y Cajal University Hospital, Madrid, Spain; 13 Colorectal Unit, Vall d´Hebrón University Hospital. Universitat Autónoma de Barcelona UAB, Barcelona, Spain; 14 Surgery Department, Alcorcon Foundation Hospital, Madrid, Spain; 15 Surgery Department, Gregorio Marañon University Hospital, Madrid, Spain; 16 Navarra University Clinic (Clínica Universidad de Navarra), Pamplona, Navarra, Spain; 17 Department of Gastroenterology, Hospital Clínic de Barcelona; Institut d’Investigacions Biomèdiques August Pi i Sunyer (IDIBAPS); Centro de Investigación Biomédica en Red de Enfermedades Hepáticas y Digestivas (CIBEREHD); University of Barcelona, Barcelona, Spain; 18 Department of Biomedical Sciences, Humanitas University, Pieve Emanuel, Milan, Italy. IRCCS Humanitas Research Hospital, Rozzano, Milan, Italy; 19 Immunogenetics Unit, National Center of Microbiology, Instituto de Salud Carlos III, Majadahonda, Madrid, Spain; 20 Biomedical Research Institute of Salamanca (IBSAL), Salamanca, Spain; 21 Coloproctology Unit, Hospital Universitario Vithas Madrid Arturo Soria, Madrid, Spain

**Keywords:** colorectal neoplasms, early diagnosis, immune response, T-cell subsets, cytokine profiling, immune biomarkers

“Affiliation Immunopathology and Viral Reservoir Unit, National Center of Microbiology, Instituto de Salud Carlos III, Majadahonda, Madrid, Spain” was omitted for “Clara Sánchez-Menéndez; Jaime Rodríguez-Pérez; Valentina Leguizamon; María González-Sanmartin; Elena Mateos; Montserrat Torres; Mayte Coiras”. This affiliation has now been added for “Clara Sánchez-Menéndez; Jaime Rodríguez-Pérez; Valentina Leguizamon; María González-Sanmartin; Elena Mateos; Montserrat Torres; Mayte Coiras”.

“Clara Sánchez-Menéndez; Jaime Rodríguez-Pérez; María González-Sanmartin; Elena Mateos; Montserrat Torres; Mayte Coiras” was erroneously assigned to affiliation: “Department of Hematology and Hemotherapy, Instituto Ramón y Cajal de Investigación Sanitaria (IRYCIS), Hospital Universitario Ramón y Cajal, Madrid, Spain”. This affiliation has now been removed for “Clara Sánchez-Menéndez; Jaime Rodríguez-Pérez; María González-Sanmartin; Elena Mateos; Montserrat Torres; Mayte Coiras”.

The original version of this article has been updated.

