# Supplementary material for: Differences in the peripheral blood immune landscape between early-onset and late-onset colorectal cancer
Source: Front Immunol. 2025 Dec 4;16:1692382. doi: 10.3389/fimmu.2025.1692382 (PMC12711750; doi:10.3389/fimmu.2025.1692382)
Supplement: Supplementary file 3 [file Presentation3.pptx]

## Slide 1
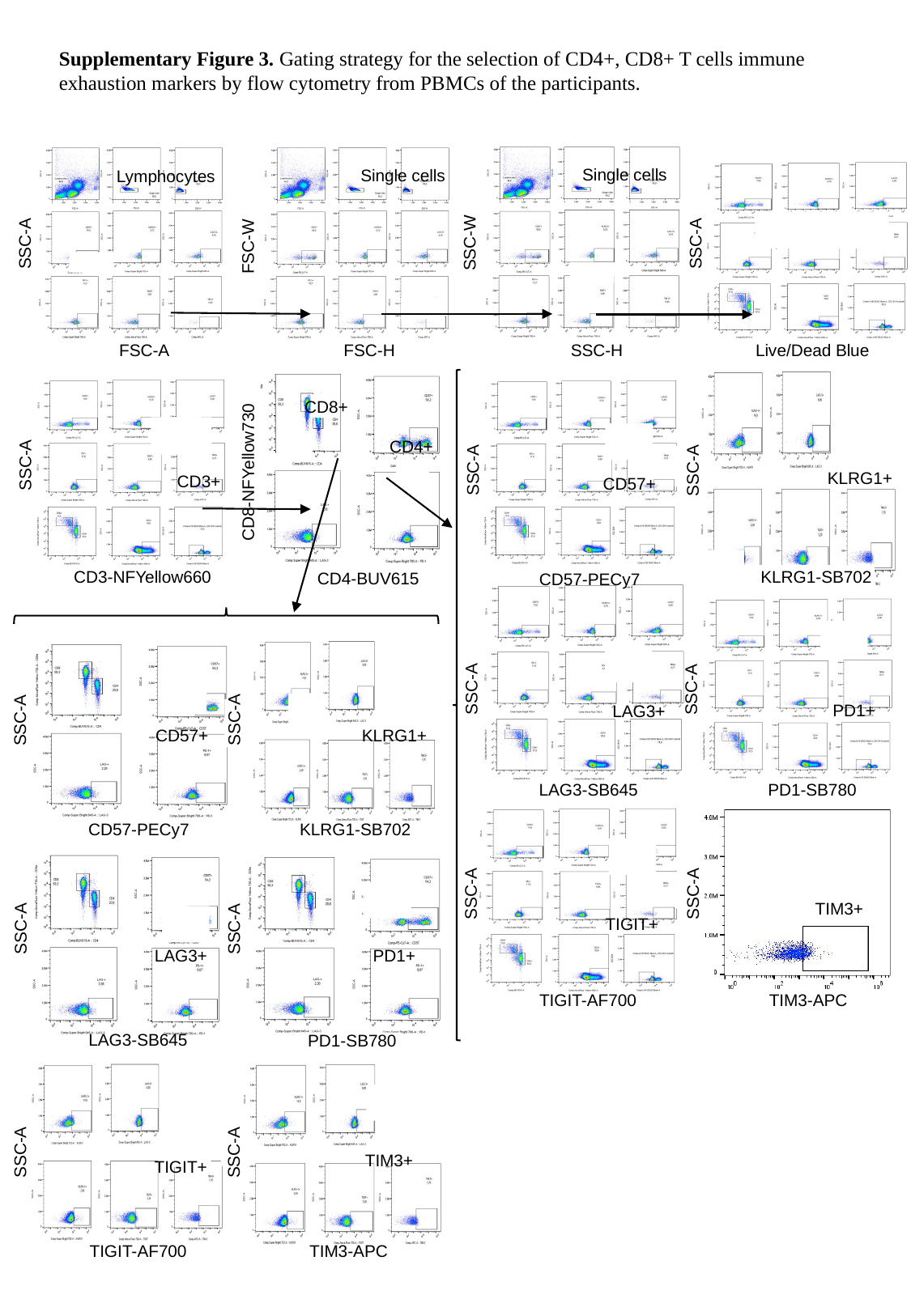

Supplementary Figure 3. Gating strategy for the selection of CD4+, CD8+ T cells immune exhaustion markers by flow cytometry from PBMCs of the participants.
Single cells
Single cells
Lymphocytes
SSC-W
SSC-A
SSC-A
FSC-W
FSC-A
FSC-H
SSC-H
Live/Dead Blue
CD8+
CD4+
SSC-A
SSC-A
SSC-A
CD8-NFYellow730
KLRG1+
CD3+
CD57+
CD3-NFYellow660
KLRG1-SB702
CD4-BUV615
CD57-PECy7
SSC-A
SSC-A
PD1+
LAG3+
SSC-A
SSC-A
CD57+
KLRG1+
LAG3-SB645
PD1-SB780
CD57-PECy7
KLRG1-SB702
SSC-A
SSC-A
TIM3+
TIGIT+
SSC-A
SSC-A
PD1+
LAG3+
TIGIT-AF700
TIM3-APC
LAG3-SB645
PD1-SB780
SSC-A
SSC-A
TIM3+
TIGIT+
TIGIT-AF700
TIM3-APC
