# Supplementary material for: Differences in the peripheral blood immune landscape between early-onset and late-onset colorectal cancer
Source: Front Immunol. 2025 Dec 4;16:1692382. doi: 10.3389/fimmu.2025.1692382 (PMC12711750; doi:10.3389/fimmu.2025.1692382)
Supplement: Supplementary file 4 [file Presentation4.pptx]

## Slide 1
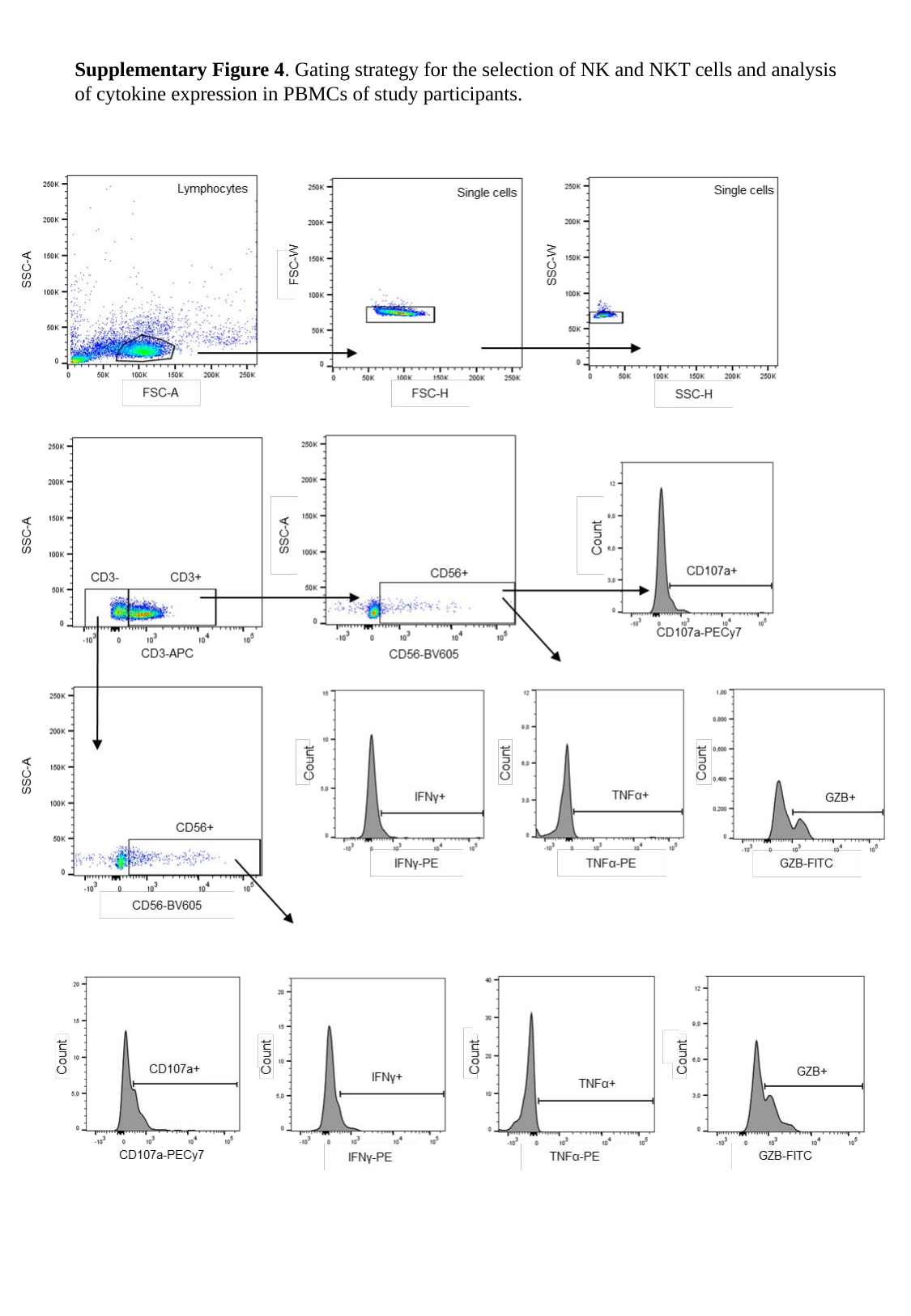

Supplementary Figure 4. Gating strategy for the selection of NK and NKT cells and analysis of cytokine expression in PBMCs of study participants.
