# Supplementary material for: Differences in the peripheral blood immune landscape between early-onset and late-onset colorectal cancer
Source: Front Immunol. 2025 Dec 4;16:1692382. doi: 10.3389/fimmu.2025.1692382 (PMC12711750; doi:10.3389/fimmu.2025.1692382)
Supplement: Supplementary file 5 [file Presentation5.pptx]

## Slide 1
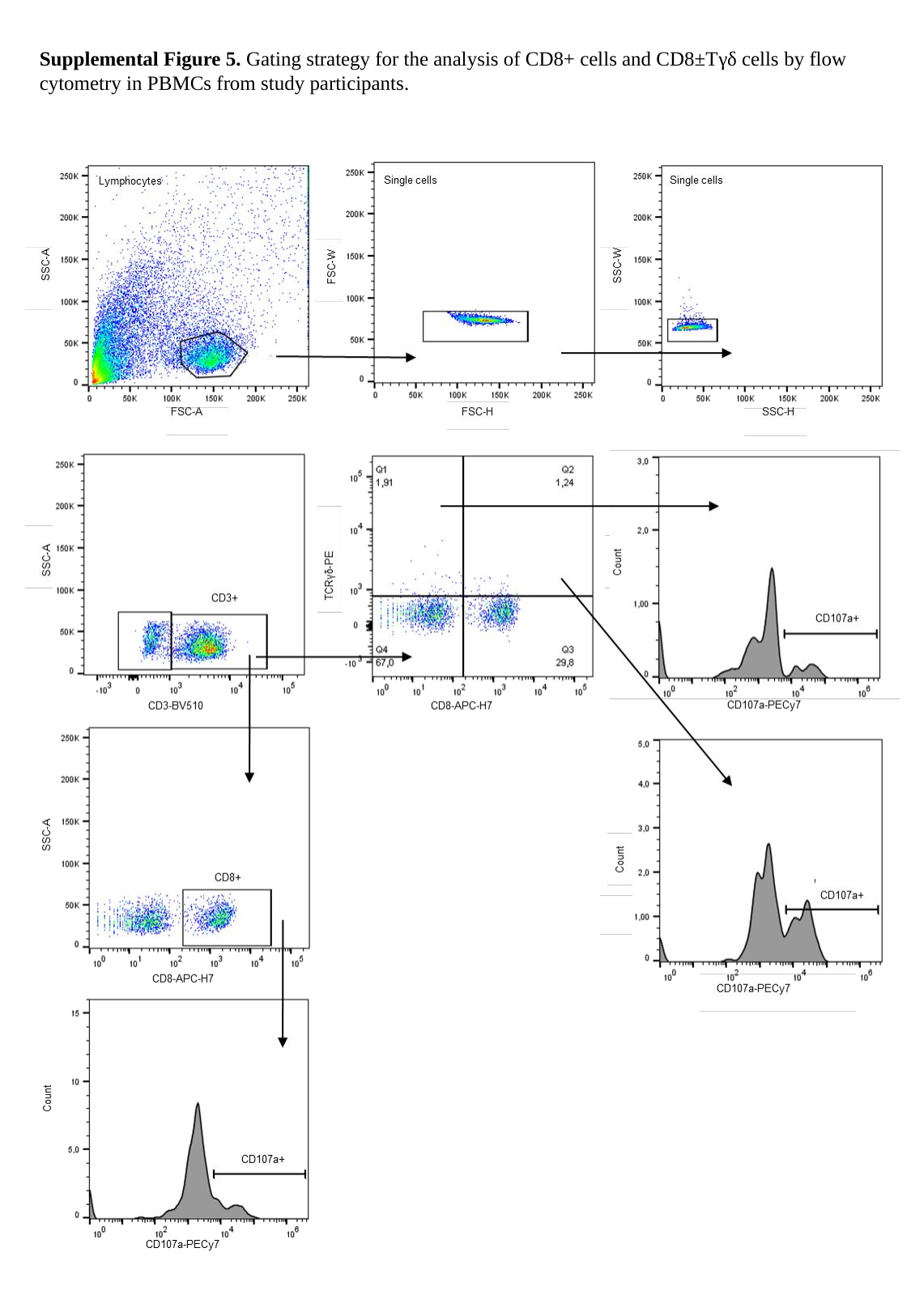

Supplemental Figure 5. Gating strategy for the analysis of CD8+ cells and CD8±Tγδ cells by flow cytometry in PBMCs from study participants.
