# Supplementary material for: Differences in the peripheral blood immune landscape between early-onset and late-onset colorectal cancer
Source: Front Immunol. 2025 Dec 4;16:1692382. doi: 10.3389/fimmu.2025.1692382 (PMC12711750; doi:10.3389/fimmu.2025.1692382)
Supplement: Supplementary file 6 [file Presentation6.pptx]

## Slide 1
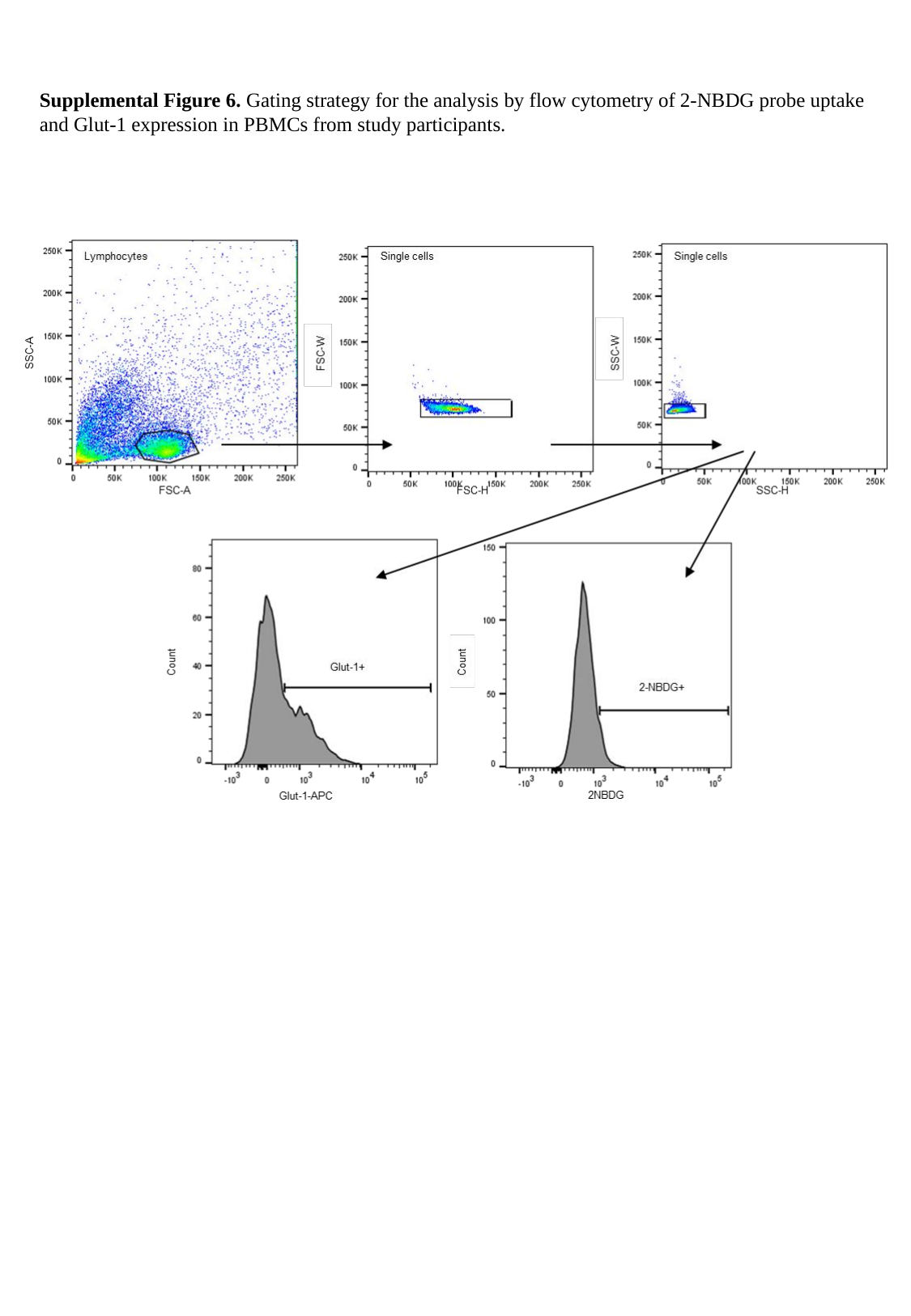

Supplemental Figure 6. Gating strategy for the analysis by flow cytometry of 2-NBDG probe uptake and Glut-1 expression in PBMCs from study participants.
