# Supplementary material for: Differences in the peripheral blood immune landscape between early-onset and late-onset colorectal cancer
Source: Front Immunol. 2025 Dec 4;16:1692382. doi: 10.3389/fimmu.2025.1692382 (PMC12711750; doi:10.3389/fimmu.2025.1692382)
Supplement: Supplementary file 7 [file Presentation7.pptx]

## Slide 1
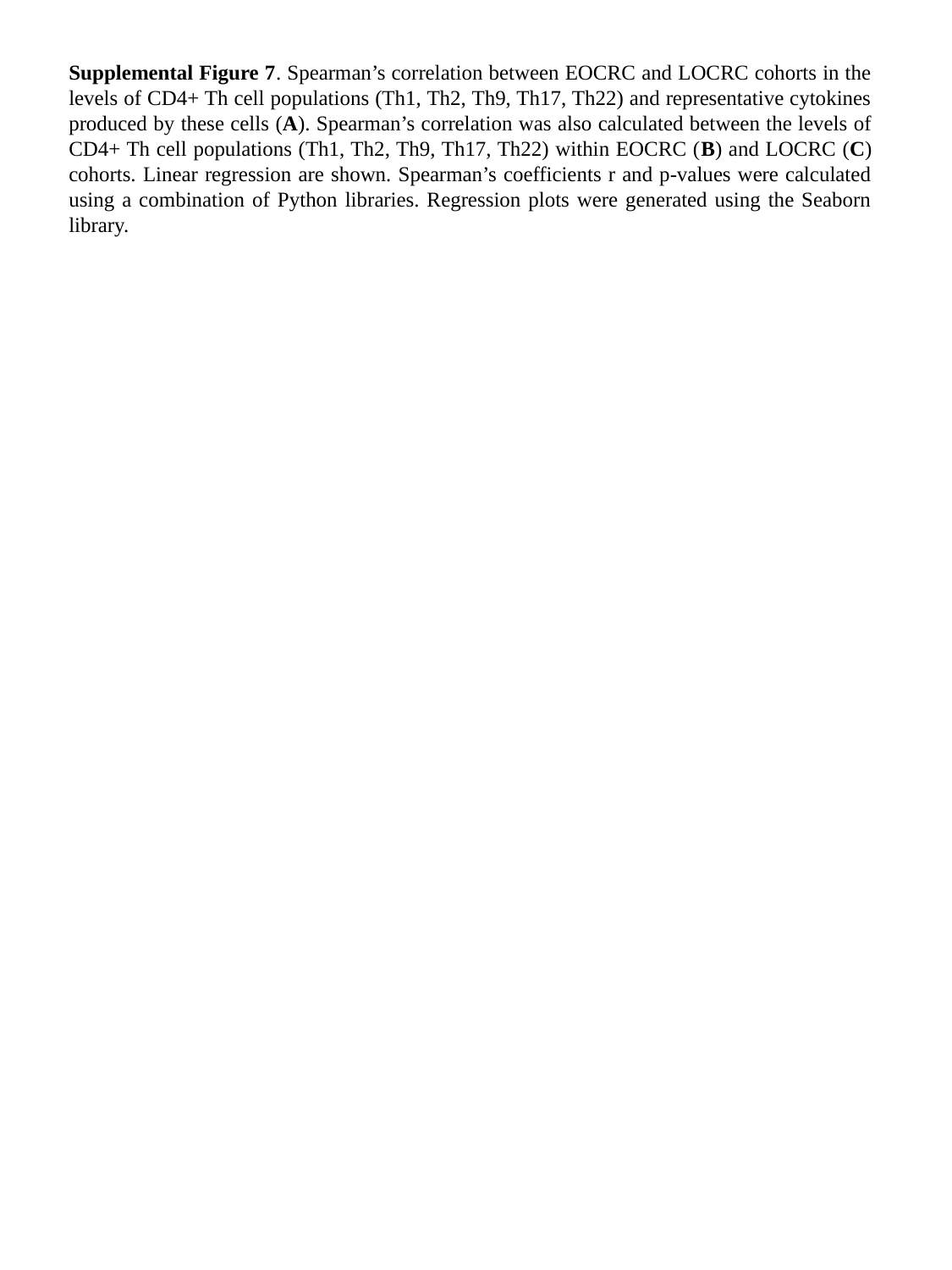

Supplemental Figure 7. Spearman’s correlation between EOCRC and LOCRC cohorts in the levels of CD4+ Th cell populations (Th1, Th2, Th9, Th17, Th22) and representative cytokines produced by these cells (A). Spearman’s correlation was also calculated between the levels of CD4+ Th cell populations (Th1, Th2, Th9, Th17, Th22) within EOCRC (B) and LOCRC (C) cohorts. Linear regression are shown. Spearman’s coefficients r and p-values were calculated using a combination of Python libraries. Regression plots were generated using the Seaborn library.

## Slide 2
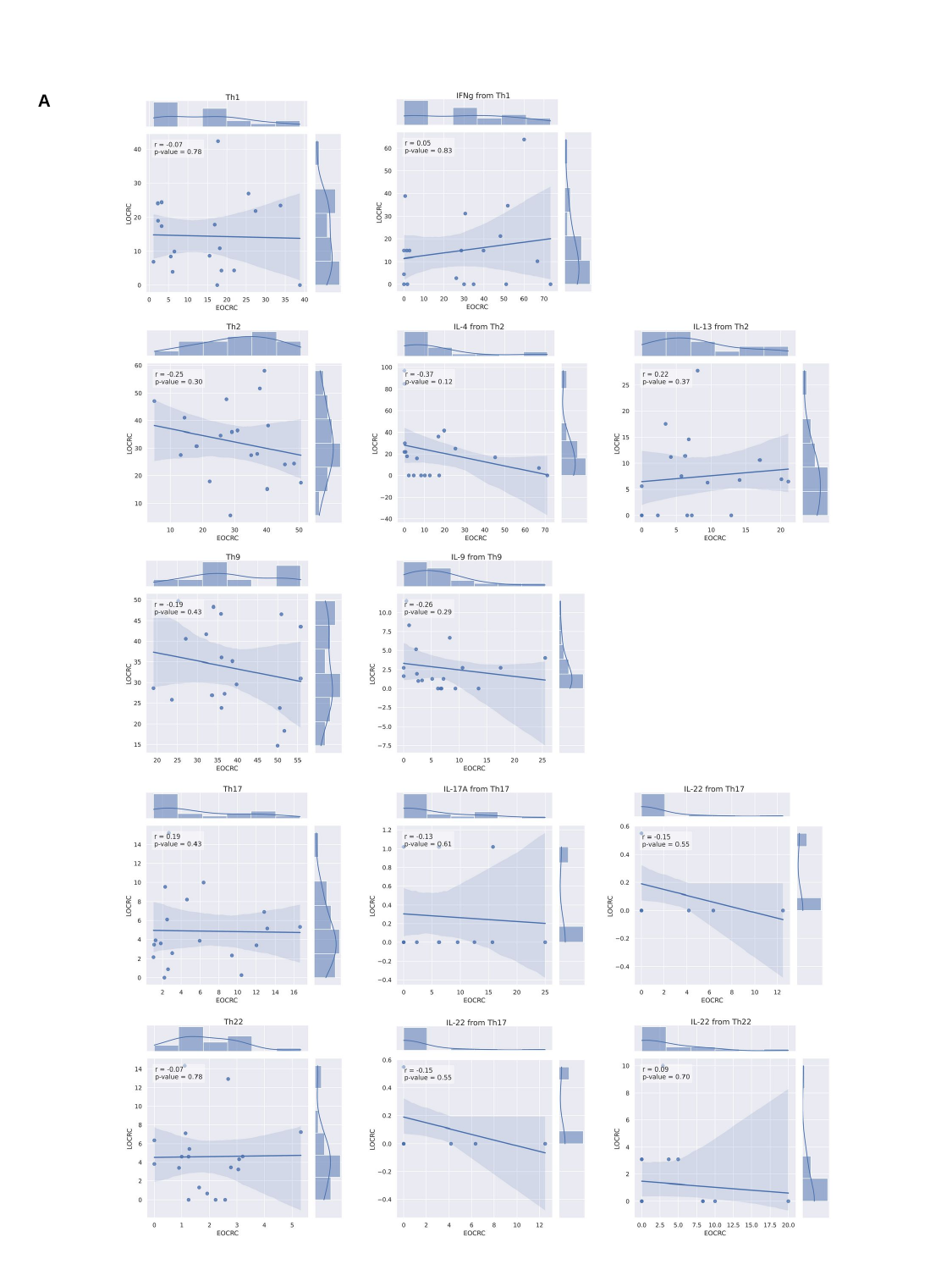

A

## Slide 3
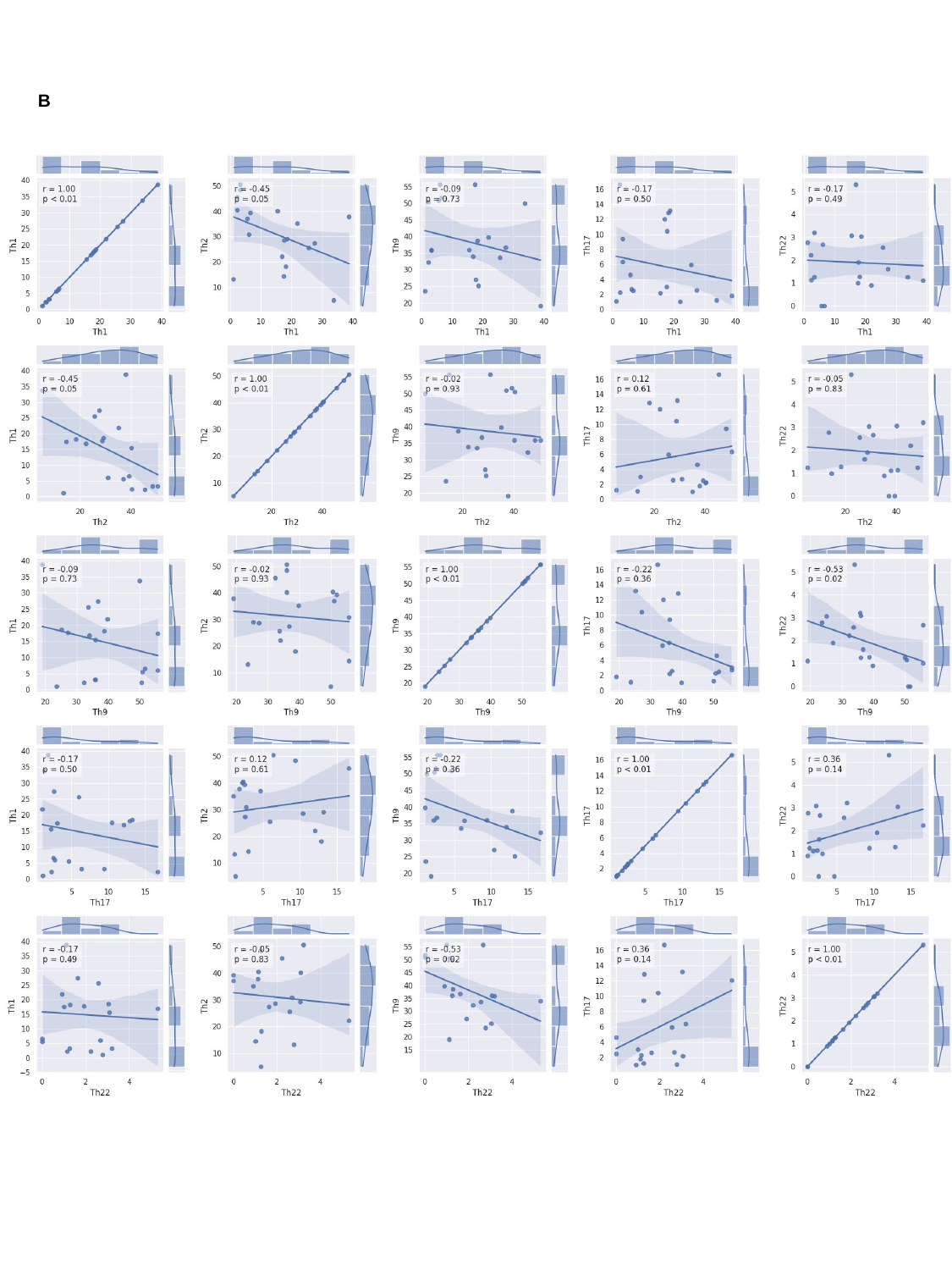

B

## Slide 4
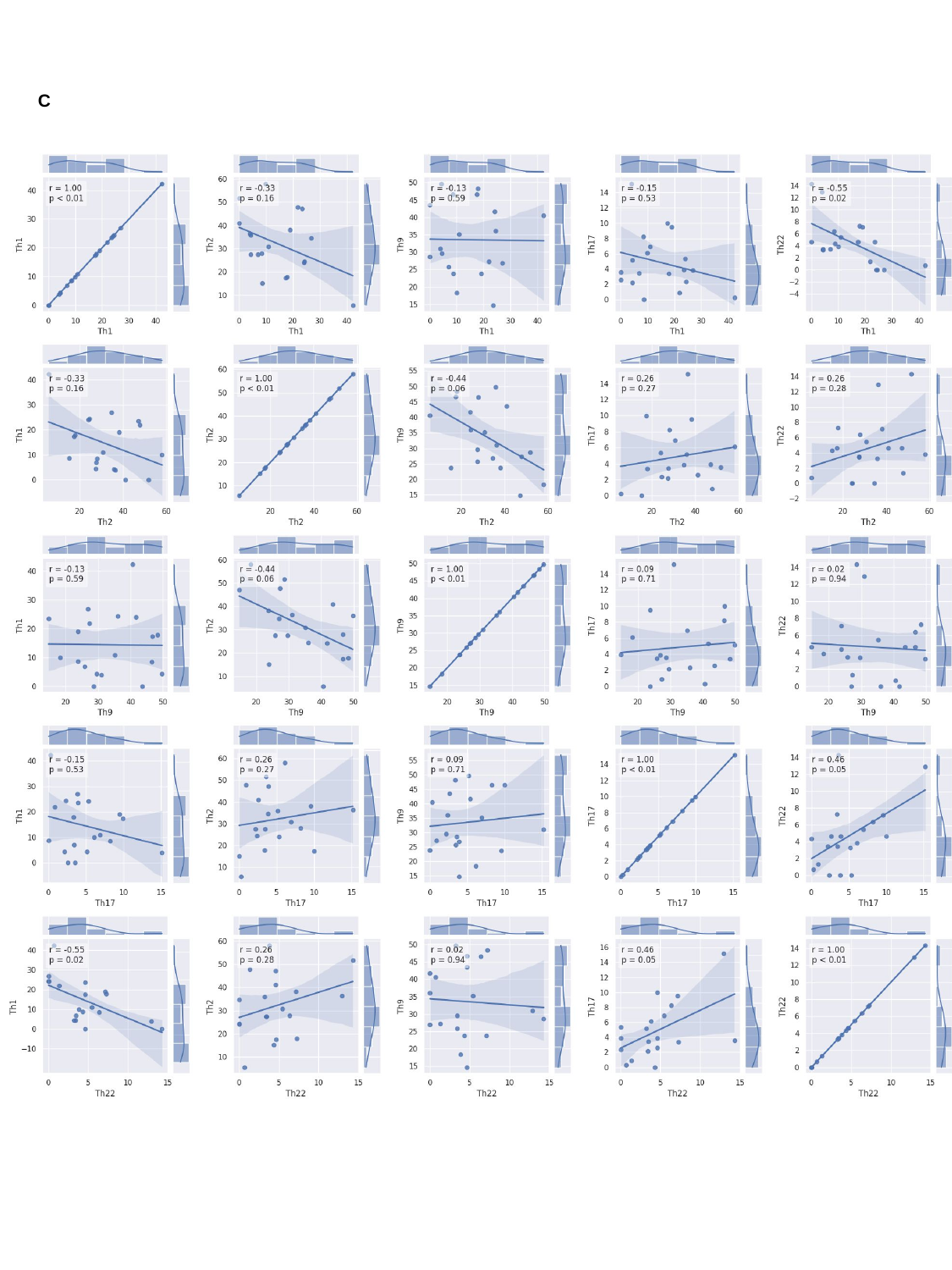

C
