# Supplementary material for: Differences in the peripheral blood immune landscape between early-onset and late-onset colorectal cancer
Source: Front Immunol. 2025 Dec 4;16:1692382. doi: 10.3389/fimmu.2025.1692382 (PMC12711750; doi:10.3389/fimmu.2025.1692382)
Supplement: Supplementary file 8 [file Presentation8.pptx]

## Slide 1
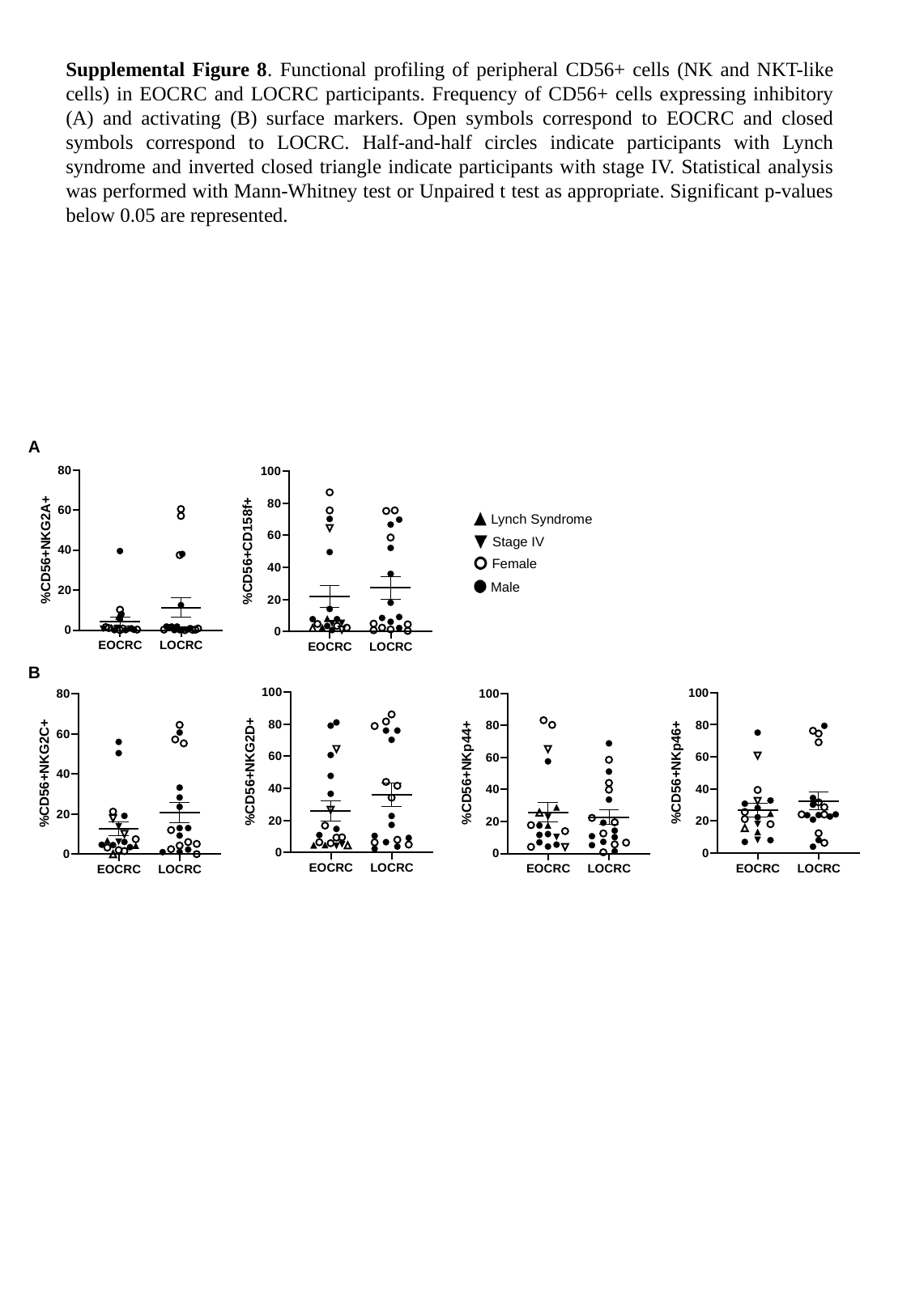

Supplemental Figure 8. Functional profiling of peripheral CD56+ cells (NK and NKT-like cells) in EOCRC and LOCRC participants. Frequency of CD56+ cells expressing inhibitory (A) and activating (B) surface markers. Open symbols correspond to EOCRC and closed symbols correspond to LOCRC. Half-and-half circles indicate participants with Lynch syndrome and inverted closed triangle indicate participants with stage IV. Statistical analysis was performed with Mann-Whitney test or Unpaired t test as appropriate. Significant p-values below 0.05 are represented.
A
B
Lynch Syndrome
Stage IV
Female
Male
