# Supplementary material for: Differences in the peripheral blood immune landscape between early-onset and late-onset colorectal cancer
Source: Front Immunol. 2025 Dec 4;16:1692382. doi: 10.3389/fimmu.2025.1692382 (PMC12711750; doi:10.3389/fimmu.2025.1692382)
Supplement: Supplementary file 9 [file Table1.docx]

**Table 1.** Sociodemographic and clinical data of all participants in the study. Fisher´s exact test was used to calculate statistical differences between cohorts. Significant p-values are highlighted in bold.

|  | **EOCRC**  **(n=19)** | **LOCRC**  **(n=19)** | **p value** |
| --- | --- | --- | --- |
| Age at cancer diagnosis, years; median (IQR) | 44 (41-48) | 76 (55-84) | **<0.0001** |
| Sex; n. male (%) | 12 (63.2) | 10 (52.6) | 0.7431 |
| Cancer location | | | |
| Right colon, n. (%) | 5 (26.4) | 6 (31.6) | 1 |
| Left colon, n. (%) | 7 (36.8) | 8 (42.1) | 1 |
| Rectum, n. (%) | 7 (36.8) | 5 (26.3) | 0.7281 |
| Stage | | | |
| I, n. (%) | 3 (15.8) | 5 (26.3) | 0.6928 |
| II, n. (%) | 5 (26.3) | 7 (36.8) | 0.7281 |
| III, n. (%) | 6 (31.6) | 7 (36.8) | 1 |
| IV, n. (%) | 5 (26.3) | 0 (0) | **0.0463** |
| Microsatellite instability, n. (%) | 3 (15.8) | 0 (0) | 0.2297 |
| CRC family history | | | |
| FDR, n. (%) | 7 (36.8) | Unk. | - |
| SDR, n. (%) | 2 (10.5) | Unk. | - |
| Sporadic, n. (%) | 10 (52.6) | Unk. | - |
| Main comorbidities | | | |
| DM, n. (%) | 2 (10.5) | 4 (21.1) | 0.6599 |
| DL, n. (%) | 2 (10.5) | 4 (21.1) | 0.6599 |
| HTN, n. (%) | 0 (0) | 10 (52.6) | **0.0004** |
| Heart conditions, n. (%) | 0 (0) | 5 (26.3) | **0.0463** |
| Respiratory conditions, n. (%) | 2 (10.5) | 4 (21.1) | 0.6599 |
| Main treatment at sample collection | | | |
| Lipid lowering medication, n. (%) | 2 (10.5) | 7 (36.8) | 0.1245 |
| Anticoagulants, n. (%) | 1 (5.3) | 7 (36.8) | **0.0422** |
| Antihypertensive medication, n. (%) | 0 (0) | 8 (42.1) | **0.0031** |
| Diabetes medication, n. (%) | 2 (10.5) | 3 (15.8) | 1 |

BPH, Benign Prostatic Hyperplasia; CRC, Colorectal cancer; DM, Diabetes mellitus; DL, Dyslipidemia; FDR, First degree relative; HTN, Hypertension; IQR, Interquartile Range; SDR, Second degree relative; Unk., Unknown.
