# Supplementary material for: Differences in the peripheral blood immune landscape between early-onset and late-onset colorectal cancer
Source: Front Immunol. 2025 Dec 4;16:1692382. doi: 10.3389/fimmu.2025.1692382 (PMC12711750; doi:10.3389/fimmu.2025.1692382)
Supplement: Supplementary file 10 [file Table2.docx]

| **Patient’s ID** | **Sex**  **(M/F)** | **Age at CRC diagnosis**  **(years)** | **Treatments**  **at sampling** | **Comorbidities** | **Cancer location** | **Stage** | **Microsatellite**  **stability/ instability** | **CRC family history** |
| --- | --- | --- | --- | --- | --- | --- | --- | --- |
| 1 | M | 44 | Pitavastatin | DL | Left colon | IV | MSS | Sporadic |
| 2 | F | 48 | No treatment | None | Rectum | I | IMS  MSH2(-), MSH6 (-) | FDR |
| 3 | M | 41 | No treatment | None | Rectum | II | IMS  MSH6 (-) | SDR |
| 4 | M | 48 | Omeprazol, salbutamol, aclidinium bromide, beclomethasone, formoterol | Asthma, bronchiectasis, DL | Left colon | III | MSS | Sporadic |
| 5 | M | 42 | No treatment | Horner syndrome | Rectum | III | MSS | FDR |
| 6 | F | 46 | No treatment | None | Right colon | III | MSS | FDR |
| 7 | F | 45 | Levocetirizine, atorvastatin | Allergy | Rectum | III | MSS | Sporadic |
| 8 | F | 49 | Sitagliptin,  oral iron supplementation | Type II DM | Rectum | I | MSS | FDR |
| 9 | F | 48 | No treatment | None | Left colon | III | MSS | Sporadic |
| 10 | F | 45 | No treatment | None | Left colon | II | MSS | Sporadic |
| 11 | M | 37 | No treatment | None | Right colon | II | IMS  MLH1(-), PMS2(-) | FDR |
| 12 | M | 44 | Enoxaparin | Bilateral pulmonary embolism | Left colon | IV | MSS | SDR |
| 13 | F | 48 | No treatment | None | Right colon | IV | MSS | Sporadic |
| 14 | M | 47 | No treatment | None | Left colon | I | MSS | Sporadic |
| 15 | F | 44 | Paliative chemotherapy | Unknown | Rectum | IV | MSS | FDR |
| 16 | M | 42 | No treatment | None | Left colon | III | MSS | Sporadic |
| 17 | M | 34 | No treatment | Migraines | Right colon | II | MSS | Sporadic |
| 18 | M | 38 | No treatment | None | Rectum | III | MSS | FDR |
| 19 | M | 30 | Insulin | Type I DM, possible celiac disease | Right colon | II | MSS | Sporadic |

**Supplemental Table 1.** Sociodemographic and clinical data of participants diagnosed with EOCRC (A) and LOCRC (B).

**A)**

CRC, Colorectal cancer; DM, Diabetes mellitus; DL, Dyslipidemia; F, Female; FDR, First degree relative; M, Male; MLH1, MutL homolog 1; MSH2, MutS homolog 2; MSH6, MutS homolog 6; MSI, microsatellite instability; MSS, microsatellite stability; PMS2, PMS1 Homolog 2, Mismatch Repair System Component; SDR, Second degree relative.

**B)**

| **Patient’s ID** | **Sex**  **(M/F)** | **Age at CRC diagnosis**  **(years)** | **Treatments**  **at sampling** | **Comorbidities** | **Cancer location** | **Stage** | **Microsatellite**  **stability/ instability** | **CRC family history** |
| --- | --- | --- | --- | --- | --- | --- | --- | --- |
| 1 | M | 82 | Pantoprazol, enoxaparin | HTN, BPH | Right (transverse) colon | III | MSS | Unknown |
| 2 | M | 80 | Mirtazapine, acetaminophen, mirabegron, tamsulosin | Amaurosis, BPH, mild dementia | Rectum | II | MSS | Unknown |
| 3 | F | 94 | Acetylsalicylic acid, torasemide, atenolol, enalapril, hydroxyzine, tramadol, acetaminophen | HTN, Bowen’s disease, carotid artery stenosis, hiatal hernia | Left colon | II | MSS | Unknown |
| 4 | F | 85 | Enalapril, simvastatin, bromazepam | HTN, DL | Rectum | II | MSS | Unknown |
| 5 | M | 67 | Carvedilol, enalapril, venlafaxine, ezetimibe, simvastatin, acetylsalicylic acid | HTN, type II DM, myocardial infarction (2 stents) | Left colon | I | MSS | Unknown |
| 6 | F | 79 | Atenolol, metformin, lisinopril, hydrochlorothiazide, gemfibrozil, simvastatin, allopurinol, ferrous sulfate, vitamin B12 | HTN, type II DM, hyperuricemia, atrial fibrillation | Right colon | II | MSS | Unknown |
| 7 | M | 84 | Salbutamol, ipratropium bromide, salbutamol, calcifediol, betamethasone, beclometasone, formoterol, glycopyrronium, brimonidine | DL, EPOC, BPH | Rectum | I | MSS | Unknown |
| 8 | M | 80 | Repaglinide, insulin, dexamethasone, budesonide, linagliptin, metformin, salbutamol, omeprazole, simvastatin, salmeterol, fluticasone | Type II DM, hypecholesterolemia, asthma | Rectum | I | MSS | Unknown |
| 9 | M | 51 | No treatment | No treatment | Left colon | II | MSS | Unknown |
| 10 | F | 76 | Apixaban, estetrol, bemiparin sodium, omeprazole, diazepam, enalapril, hydrochlorothiazide | HTN, DL, pulmonary embolism, breast and uterine cancer | Left colon | II | MSS | Unknown |
| 11 | F | 67 | Levothyroxine, olmesartan, rosuvastatin, duloxetine, diazepam | HTN, DL, obesity, hypothyroidism, fibromyalgia, penicillin allergy | Right colon | I | MSS | Unknown |
| 12 | F | 55 | No treatment | Smoker | Left colon | II | MSS | Unknown |
| 13 | M | 74 | Apixaban, bisoprolol, amlodipine, enalapril | HTN, atrial fibrillation, ischemic cardiomyopathy, daily drinker | Left colon | III | MSS | Unknown |
| 14 | M | 88 | No treatment | Hip osteoarthritis | Right colon | I | MSS | Unknown |
| 15 | M | 54 | Ferrous sulfate, linagliptin, levothyroxine, clonazepam, vortioxetine, enalapril, tramadol, acetaminophen, metamizole, simvastatin, baclofen, metformin, calcifediol, acetaminophen, propranolol, atorvastatin, captopril, bemiparin sodium, esomeprazole, amlodipine, acetylsalicylic acid, levetiracetam | Type II DM, schizophrenia, hypercholesterolemia, HTN, brain hemorrhage, left hemiparesis, thyroiditis due to COVID-19 | Right colon | III | MSS | Unknown |
| 16 | F | 52 | Lorazepam, metamizole | Anxiety | Right colon | II | MSS | Unknown |
| 17 | F | 87 | Amiodarone, edoxaban | Sinus node dysfunction | Rectum | III | MSS | Unknown |
| 18 | M | 53 | No treatment | HTN, past hepatitis C | Left colon | I | MSS | Unknown |
| 19 | F | 56 | No treatment | No treatment | Left colon | III | MSS | Unknown |

BPH, Benign Prostatic Hyperplasia; CRC, Colorectal cancer; DM, Diabetes mellitus; DL, Dyslipidemia; F, Female; FDR, First degree relative; HTN, Hypertension; M, Male; MLH1, MutL homolog 1; MSH2, MutS homolog 2; MSH6, MutS homolog 6; MSI, microsatellite instability; MSS, microsatellite stability; PMS2, PMS1 Homolog 2, Mismatch Repair System Component; SDR, Second degree relative.
