# Supplementary material for: Differences in the peripheral blood immune landscape between early-onset and late-onset colorectal cancer
Source: Front Immunol. 2025 Dec 4;16:1692382. doi: 10.3389/fimmu.2025.1692382 (PMC12711750; doi:10.3389/fimmu.2025.1692382)
Supplement: Supplementary file 11 [file Table3.docx]

| Variable | Binary logistic regression | | |
| --- | --- | --- | --- |
|  | **OR** | **95% CI** | **p-value** |
| Sex |  |  |  |
| Female | 1.2375 | 0.3438 to 4.4543 | 0.744 |
| Cancer location (Ref. Right) |  |  |  |
| Left colon | 1.1429 | 0.2303 to 5.6704 | 0.870 |
| Rectum | 0.7143 | 0.1319 to 3.8683 | 0.696 |
| Cancer stage (Ref. Stage I) |  |  |  |
| Stage II | 0.8000 | 0.1349 to 4.7450 | 0.806 |
| Stage III | 0.3571 | 0.0591 to 2.1593 | 0.262 |
| Stage IV | 1 | - | - |
| Comorbidities (Ref. No comorbidity) |  |  |  |
| DL | 4.2500 | 0.7292 to 24.7693 | 0.108 |
| HTN | 1 | - | - |
| DM | 2.2667 | 0.3622 to 14.1852 | 0.382 |
| Heart conditions | 1 | - | - |
| Respiratory conditions | 1.5938 | 0.2348 to 10.8168 | 0.633 |
| Treatment at sample (Ref. No treatment) |  |  |  |
| Lipid-lowering medication | 4.9583 | 0.8733 to 28.1520 | 0.071 |
| Anticoagulants | **10.5000** | **1.1416 to 96.5763** | **0.038** |
| Blood pressure medication | 1 | - | - |
| Diabetes medication | 1.5938 | 0.2348 to 10.8168 | 0.633 |

**Supplemental Table 2.** Binary logistic regression analysis predicting the development of CRC in the participants in the study. Significant p-values are highlighted in bold.

95% CI, 95% confidence interval; DL, Dyslipidemia; DM, Diabetes mellitus; HTN, hypertension; OR, Odds Ratio; Ref., Reference parameter.
