# Supplementary material for: Differences in the peripheral blood immune landscape between early-onset and late-onset colorectal cancer
Source: Front Immunol. 2025 Dec 4;16:1692382. doi: 10.3389/fimmu.2025.1692382 (PMC12711750; doi:10.3389/fimmu.2025.1692382)
Supplement: Supplementary file 12 [file Table4.docx]

**Supplemental Table 3.** Associations between immune parameters analyzed in cells (A) and plasma (B) and the age of CRC development were assessed using simple linear regression analysis and subsequent binary logistic regression analysis (OR). Significant p-values are highlighted in bold.

**A)**

| **Variable** | **β** | **Simple linear 95% CI** | **p-value** | **OR** | **Binary logistic 95% CI** | **p-value** |
| --- | --- | --- | --- | --- | --- | --- |
| Age at diagnosis | 0.0227 | 0.0171 to 0.0283 | **<0.001** | - | - | - |
| CD3+ | -0.0082 | -0.0165 to 0.0001 | 0.053 | 0.9634 | 0.9255 to 1.0028 | 0.068 |
| CD4+ | 0.0069 | -0.0029 to 0.0167 | 0.162 | 1.0294 | 0.9883 to 1.0723 | 0.164 |
| Tregs | -0.0084 | -0.0351 to 0.0183 | 0.528 | 0.9668 | 0.8729 to 1.0708 | 0.517 |
| CD4+ CD57+ | -0.0102 | -0.0225 to 0.0021 | 0.100 | 0.8851 | 0.7430 to 1.0545 | 0.172 |
| CD4+ KLRG1+ | -0.0197 | -0.0692 to 0.0299 | 0.426 | 0.9214 | 0.7547 to 1.1248 | 0.421 |
| CD4+ LAG3+ | -0.0180 | -0.0658 to 0.0298 | 0.448 | 0.9287 | 0.7705 to 1.1195 | 0.438 |
| CD4+ PD1+ | -0.0269 | -0.0579 to 0.0041 | 0.087 | 0.8637 | 0.7204 to 1.0356 | 0.114 |
| CD4+ TIGIT+ | 0.0187 | -0.0143 to 0.0517 | 0.257 | 1.0835 | 0.9424 to 1.2457 | 0.260 |
| CD4+ TIM3+ | -0.0412 | -0.1213 to 0.0389 | 0.304 | 0.8428 | 0.6096 to 1.1652 | 0.301 |
| CD4+ Th1 cells | -0.0008 | -0.0163 to 0.0147 | 0.918 | 0.9968 | 0.9403 to 1.0568 | 0.915 |
| IFNγ from Th1 | -0.0064 | -0.0138 to 0.0009 | 0.084 | 0.9718 | 0.9401 to 1.0045 | 0.091 |
| CD4+ Th2 cells | 0.0016 | -0.0117 to 0.0149 | 0.809 | 1.0064 | 0.9570 to 1.0584 | 0.803 |
| IL4 from Th2 | 0.0022 | -0.0047 to 0.0091 | 0.517 | 1.0090 | 0.9826 to 1.0362 | 0.508 |
| IL13 from Th2 | 0.0039 | -0.0151 to 0.0230 | 0.677 | 1.0162 | 0.9443 to 1.0936 | 0.668 |
| CD4+ Th9 cells | -0.0106 | -0.0258 to 0.0046 | 0.167 | 0.9570 | 0.8994 to 1.0184 | 0.166 |
| IL9 from Th9 | -0.0314 | -0.0611 to -0.0017 | **0.039** | 0.8368 | 0.6956 to 1.0067 | 0.059 |
| CD4+ Th17 cells | -0.0134 | -0.0522 to 0.0254 | 0.487 | 0.9470 | 0.8151 to 1.1003 | 0.477 |
| IL17A from Th17 | -0.0240 | -0.0515 to 0.0034 | 0.083 | 0.8678 | 0.7252 to 1.0385 | 0.122 |
| IL22 from Th17 | -0.0075 | -0.0225 to 0.0075 | 0.314 | 0.8819 | 0.6482 to 1.1999 | 0.424 |
| CD4+ Th22 cells | 0.0638 | 0.0142 to 0.1134 | **0.013** | 1.5012 | 1.0321 to 2.1836 | **0.034** |
| IL13 from Th22 | -0.0250 | -0.0468 to -0.0032 | **0.026** | 0.8835 | 0.7832 to 0.9967 | **0.044** |
| IL22 from Th22 | -0.0058 | -0.0244 to 0.0128 | 0.529 | 0.9741 | 0.8984 to 1.0561 | 0.524 |
| K562 Apoptosis | 0.0159 | -0.0122 to 0.0439 | 0.259 | 1.0722 | 0.9475 to 1.2133 | 0.269 |
| CD8+ | 0.0014 | -0.0078 to 0.0105 | 0.765 | 1.0055 | 0.9713 to 1.0408 | 0.758 |
| CD8+ CD107a+ | -0.0048 | -0.0206 to 0.0111 | 0.545 | 0.9805 | 0.9216 to 1.0433 | 0.535 |
| CD8+ CD57+ | -0.0009 | -0.0096 to 0.0079 | 0.845 | 0.9966 | 0.9640 to 1.0303 | 0.839 |
| CD8+ KLRG1+ | 0.0021 | -0.0151 to 0.0193 | 0.806 | 1.0085 | 0.9445 to 1.0768 | 0.800 |
| CD8+ LAG3+ | -0.0403 | -0.0857 to 0.0051 | 0.080 | 0.8336 | 0.6741 to 1.0307 | 0.093 |
| CD8+ PD1+ | -0.0225 | -0.0484 to 0.0034 | 0.087 | 0.9073 | 0.8096 to 1.0169 | 0.094 |
| CD8+ TIGIT+ | -0.0191 | -0.0507 to 0.0126 | 0.229 | 0.9224 | 0.8083 to 1.0524 | 0.230 |
| CD8+ TIM3+ | -0.0079 | -0.1120 to 0.0962 | 0.879 | 0.9690 | 0.6550 to 1.4333 | 0.875 |
| CD8-TCRγδ+ | 0.0037 | -0.0329 to 0.0404 | 0.837 | 1.0151 | 0.8845 to 1.1649 | 0.831 |
| CD8-TCRγδ+ CD107a+ | 0.0060 | -0.0132 to 0.0251 | 0.530 | 1.0245 | 0.9520 to 1.1026 | 0.518 |
| CD8+ TCRγδ+ | 0.0479 | 0.0138 to 0.0820 | **0.007** | 1.4861 | 1.0289 to 2.1466 | **0.035** |
| CD8+ TCRγδ+ CD107a+ | 0.0030 | -0.0175 to 0.0235 | 0.765 | 1.0123 | 0.9374 to 1.0933 | 0.755 |
| CD3-CD56+ | 0.0071 | -0.0022 to 0.0164 | 0.128 | 1.0306 | 0.9911 to 1.0718 | 0.131 |
| CD3-CD56+ CD107a+ | 0.0128 | -0.0288 to 0.0032 | 0.114 | 0.9387 | 0.8649 to 1.0187 | 0.130 |
| CD3-CD56+IFNγ | -0.0003 | -0.0060 to 0.0055 | 0.931 | 0.9990 | 0.9776 to 1.0209 | 0.928 |
| CD3-CD56+ TNFα | -0.0022 | -0.0097 to 0.0053 | 0.553 | 0.9905 | 0.9602 to 1.0217 | 0.545 |
| CD3-CD56+ GZB | 0.0065 | -0.0002 to 0.0131 | 0.057 | 1.0287 | 0.9980 to 1.0603 | 0.068 |
| CD3+CD56+ | -0.0060 | -0.0106 to -0.0014 | **0.012** | 0.9722 | 0.9495 to 0.9954 | **0.019** |
| CD3+CD56+ CD107a+ | 0.0017 | -0.0102 to 0.0135 | 0.777 | 1.0068 | 0.9625 to 1.0531 | 0.768 |
| CD3+CD56+ IFNγ | -0.0024 | -0.0094 to 0.0046 | 0.487 | 0.9897 | 0.9626 to 1.0179 | 0.475 |
| CD3+CD56+ TNFα | -0.0017 | -0.0098 to 0.0065 | 0.683 | 0.9930 | 0.9609 to 1.0261 | 0.673 |
| CD3+CD56+ GZB | 0.0008 | -0.0084 to 0.0099 | 0.867 | 1.0032 | 0.9675 to 1.0403 | 0.861 |
| CD56+ NKG2A | 0.0069 | -0.0037 to 0.0174 | 0.194 | 1.0321 | 0.9819 to 1.0848 | 0.214 |
| CD56+ NKG2C | 0.0054 | -0.0031 to 0.0140 | 0.208 | 1.0231 | 0.9872 to 1.0604 | 0.210 |
| CD56+ NKG2D | 0.0029 | -0.0028 to 0.0086 | 0.313 | 1.0118 | 0.9895 to 1.0346 | 0.304 |
| CD56+ NKG2A+ NKG2C+ | 0.0135 | -0.0004 to 0.0274 | 0.056 | 1.3694 | 0.1514 to 12.3828 | 0.780 |
| CD56+ CD158f | 0.0016 | -0.0042 to 0.0074 | 0.583 | 1.0064 | 0.9844 to 1.0290 | 0.572 |
| CD56+ NKp44 | -0.0015 | -0.0089 to 0.0060 | 0.693 | 0.9942 | 0.9665 to 1.0226 | 0.684 |
| CD56+ NKp46+ | 0.0034 | -0.0048 to 0.0115 | 0.411 | 1.0139 | 0.9817 to 1.0472 | 0.402 |
| 2NBDG | -0.0123 | -0.0305 to 0.0058 | 0.176 | 0.9454 | 0.8686 to 1.0290 | 0.194 |
| GLUT-1 | 0.0017 | -0.0061 to 0.0095 | 0.660 | 1.0069 | 0.9776 to 1.0370 | 0.650 |

**B)**

| **Variable** | **β** | **Simple linear 95% CI** | **p-value** | **OR** | **Binary logistic 95% CI** | **p-value** |
| --- | --- | --- | --- | --- | --- | --- |
| IL1A | -0.0087 | -0.0255 to 0.0081 | 0.302 | 0.9557 | 0.8695 to 1.0504 | 0.348 |
| IL4 | -0.0011 | -0.0030 to 0.0008 | 0.260 | 0.9948 | 0.9853 to 1.0044 | 0.288 |
| IL6 | -0.0008 | -0.0159 to 0.0143 | 0.917 | 0.9969 | 0.9419 to 1.0551 | 0.914 |
| IL17 | -0.0024 | -0.0065 to 0.0017 | 0.237 | 1 | - | - |
| TNFα | -0.0046 | -0.0175 to 0.0082 | 0.467 | 0.9807 | 0.9306 to 1.0334 | 0.465 |
| IFNβ | -0.0186 | -0.0481 to 0.0110 | 0.211 | 0.8174 | 0.5028 to 1.3289 | 0.416 |
| IFNγ | -0.0006 | -0.0011 to 0.0000 | 0.062 | 0.9720 | 0.9338 to 1.0116 | 0.163 |
| IL1RA | 0.0001 | -0.0001 to 0.0002 | 0.209 | 1.0004 | 0.9998 to 1.0010 | 0.212 |
| IL10 | 0.0102 | -0.0109 to 0.0313 | 0.332 | 1.0458 | 0.9538 to 1.1467 | 0.341 |
| CCL2 | 0.0003 | -0.0014 to 0.0020 | 0.710 | 1.0013 | 0.9948 to 1.0078 | 0.702 |
| CCL3 | -0.0018 | -0.0045 to 0.0010 | 0.206 | 0.9920 | 0.9793 to 1.0049 | 0.223 |
| CCL4 | -0.0005 | -0.0012 to 0.0003 | 0.223 | 0.9978 | 0.9942 to 1.0015 | 0.242 |
| CXCL8/IL8 | -0.0126 | -0.0228 to 0.0025 | **0.016** | 0.9108 | 0.8294 to 1.0003 | 0.051 |
| IL2 | -0.0014 | -0.0057 to 0.0028 | 0.495 | 0.9939 | 0.9767 to 1.0114 | 0.494 |
| IL7 | -0.0068 | -0.0183 to 0.0047 | 0.237 | 0.9679 | 0.9136 to 1.0254 | 0.267 |
| IL15 | -0.0055 | -0.0206 to 0.0097 | 0.470 | 0.9757 | 0.9107 to 1.0453 | 0.483 |
